# Auditory Threshold Variability in the SAMP8 Mouse Model of Age-Related Hearing Loss: Functional Loss and Phenotypic Change Precede Outer Hair Cell Loss

**DOI:** 10.3389/fnagi.2021.708190

**Published:** 2021-08-02

**Authors:** Barbara Peixoto Pinheiro, Youssef Adel, Marlies Knipper, Marcus Müller, Hubert Löwenheim

**Affiliations:** ^1^Translational Hearing Research, Tübingen Hearing Research Center, Department of Otolaryngology, Head and Neck Surgery, University of Tübingen, Tübingen, Germany; ^2^Molecular Physiology of Hearing, Tübingen Hearing Research Center, Department of Otolaryngology, Head and Neck Surgery, University of Tübingen, Tübingen, Germany

**Keywords:** SAMP8 strain, age-related hearing loss, auditory brainstem response, cytocochleogram, KCNQ4 (Kv7.4), KCNQ1 (Kv7.1)

## Abstract

Age-related hearing loss (ARHL) is the most common sensory deficit in aging society, which is accompanied by increased speech discrimination difficulties in noisy environments, social isolation, and cognitive decline. The audiometric degree of ARHL is largely correlated with sensory hair cell loss in addition to age-related factors not captured by histopathological analysis of the human cochlea. Previous studies have identified the senescence-accelerated mouse prone strain 8 (SAMP8) as a model for studying ARHL and age-related modifications of the cochlear redox environment. However, the SAMP8 population exhibits a large variability in auditory function decline over age, whose underlying cause remains unknown. In this study, we analyzed auditory function of SAMP8 mice by measuring auditory brainstem response (ABR) thresholds at the age of 6 weeks (juvenile), 12 weeks (young adult), and 24 weeks (adult). Consistent with previous studies, SAMP8 mice exhibit an early progressive, age-related decline of hearing acuity. However, a spatiotemporal cytohistological analysis showed that the significant increase in threshold variability was not concurrently reflected in outer hair cell (OHC) loss observed in the lower and upper quartiles of the ABR threshold distributions over age. This functional loss was found to precede OHC loss suggesting that age-related phenotypic changes may be contributing factors not represented in cytohistological analysis. The expression of potassium channels KCNQ4 (K_V_7.4), which mediates the current I_K,n_ crucial for the maintenance of OHC membrane potential, and KCNQ1 (K_V_7.1), which is an essential component in potassium circulation and secretion into the endolymph generating the endocochlear potential, showed differences between these quartiles and age groups. This suggests that phenotypic changes in OHCs or the stria vascularis due to variable oxidative deficiencies in individual mice may be predictors of the observed threshold variability in SAMP8 mice and their progressive ARHL. In future studies, further phenotypic predictors affected by accumulated metabolic challenges over age need to be investigated as potentially underlying causes of ARHL preceding irreversible OHC loss in the SAMP8 mouse model.

## Introduction

Age-related hearing loss (ARHL), or presbycusis, is the most common sensory impairment (Bowl and Dawson, 2019). According to the comprehensive Global Burden of Disease study, hearing loss is the third most common cause of years lived with disability, with ARHL ascertained as the leading impairment in the population older than 70 years (GBD Hearing Loss Collaborators, 2021). ARHL typically presents as a decline in hearing ability with aging that is symmetrical and pronounced in the high frequencies. There is considerable variation in the age of onset, the grade of hearing loss, and the progression of the disease (Merchant and Nadol, 2010). Age-dependent loss of hearing sensitivity is accompanied by reduced speech discrimination, decelerated central processing, and impaired sound localization (Frisina and Frisina, 1997; Frisina, 2001; Merchant and Nadol, 2010). Although this condition is not considered life-threatening, it can significantly degrade the quality of life and is associated with psychological and medical morbidity, including social isolation, depression, and cognitive decline (Lin et al., 2011a, 2013; Kamil et al., 2016; Goman and Lin, 2018; Rutherford et al., 2018; Bowl and Dawson, 2019; GBD Hearing Loss Collaborators, 2021).

ARHL has been suggested to result from age-related cochlear degeneration caused by cumulative environmental effects in industrialized societies, such as noise exposure, which are not present in ethnologically isolated populations (Hilke and Plester, 1955; Rosen et al., 1962, 1964). Controlling for population-specific genetic factors, true or intrinsic presbycusis was demonstrated in other non-industrial populations (Goycoolea et al., 1986). This supports observations that ARHL develops from the multifactorial interaction of environmental, monogenic, or polygenic factors that shape a complex disease situation (Van Eyken et al., 2007; Vuckovic et al., 2018). The classic human pathophysiological model of ARHL, also known as Schuknecht’s typology (Ohlemiller, 2004), is derived from studies of audiometric tests and serial histological sections of post-mortem human temporal bones over a span of more than five decades (Schuknecht, 1955, 1964, 1974, 1993; Schuknecht and Gacek, 1993; Merchant and Nadol, 2010). According to this model, ARHL has been classified into sensory, strial, and neural presbycusis, or combinations thereof which are termed mixed presbycusis or an indeterminate type (Merchant and Nadol, 2010). A recent, modified re-analysis of human temporal bones from the same archival temporal bone collection showed that the degree of ARHL is best predicted by the loss of outer hair cells (OHCs) and inner hair cells (IHCs), strongly suggesting sensory presbycusis as the predominant type of ARHL in humans (Wu et al., 2020). This quantitative re-analysis of serial sections confirmed earlier findings using microdissected sensory epithelia that were analyzed using the surface specimen technique to generate cytocochleograms of the human cochlea (Engström et al., 1966). This technique demonstrated similar degeneration patterns of OHCs and IHCs pursuant to ARHL (Bredberg, 1968). However, in addition to sensory hair cell loss, it is proposed that other age-related detrimental effects causing phenotypic change of surviving hair cells can contribute to ARHL and which remain unexplained (Wu et al., 2020).

Increasing evidence proposes oxidative stress as one of the major risk factors for ARHL (Menardo et al., 2012; Han and Someya, 2013; Benkafadar et al., 2019). Oxidative stress refers to the imbalance between cellular production of reactive oxygen species (ROS) and antioxidant defenses (Halliwell, 1992). It can result in cell damage by oxidization of cellular components such as membrane lipids, proteins, and DNA (Serrano and Klann, 2004). Accumulation of ROS production and impaired antioxidant defense systems, e.g., due to aging stress or following noise overstimulation, are implicated to cause cellular dysfunction such as lipid peroxidation and enzyme inactivation, leading to permanent apoptotic cell degeneration and hence initiating the process of cochlear senescence (Harman, 1956; Beckman and Ames, 1998; Baker and Staecker, 2012; Fujimoto and Yamasoba, 2019). ROS-mediated modification of ion channels can also alter the activity of other signaling mechanisms leading to changes in channel activity or channel gene expression (Matalon et al., 2003). ROS overproduction can, therefore, metabolically stress the cochlea and induce mitochondrial dysfunction and an associated decrease of energy production (Balaban et al., 2005; Henderson et al., 2006; Lin and Beal, 2006). Consequently, sensitive stages that require high energy supply, e.g., potassium ion (K^+^) channels, can be severely challenged by the accumulation of ROS.

K^+^ channels are critically dependent on continuous recycling processes for proper membrane expression, suggesting them as one of the primary targets of ROS accumulation (Peixoto Pinheiro et al., 2020). In the inner ear, physiological functions of various K^+^ channels involve the adjustment of the resting potential, shaping the responses of sensory hair cells and neurons (Housley and Ashmore, 1992; Santos-Sacchi, 1993; Kros et al., 1998), synaptic inhibition in hair cells (Fuchs and Murrow, 1992; Oliver et al., 2000), and the generation of the endocochlear potential (EP) by the stria vascularis (SV) establishing a high K^+^ concentration in the endolymph (Wangemann, 2002). A number of K^+^ channels, including voltage-gated (K_V_) channels, have been shown to be modified both *in vivo* and *in vitro* by oxidants (Ruppersberg et al., 1991; Cai and Sesti, 2009). Interventions intended to either increase antioxidant activity or decrease ROS production could, therefore, be effective therapeutic approaches to prevent or decelerate ARHL. In this endeavor, animal models have been used to investigate whether restoration of normal ROS balance with antioxidants may have therapeutic value (Ohinata et al., 2003; Hamernik et al., 2008; Fetoni et al., 2009; Fujimoto and Yamasoba, 2019). For example, a recent study showed that treatment with a synthetic superoxide dismutase/catalase-induced mitigation of excessive ROS could prevent accelerated ARHL and sensory hair cell loss in the senescence-accelerated mouse prone strain 8 (SAMP8) (Benkafadar et al., 2019), a mouse model which has been introduced for studying the pathophysiology of senescence in different systems.

The SAMP8 is a mouse strain established through phenotypic selection toward accelerated senescence from the AKR/J strain (Takeda et al., 1981). SAMP8 mice have been identified as a suitable model to study age-dependent disorders including senile amyloidosis, osteoporosis, cataracts, and brain atrophies (Akiguchi et al., 2017; Karuppagounder et al., 2017; Folch et al., 2018; Grinan-Ferre et al., 2018). Furthermore, the SAMP8 strain has previously shown to exhibit an early age-dependent hearing loss (Marie et al., 2017) associated with sensory, neural, and strial degeneration, which were primarily linked to oxidative stress (Menardo et al., 2012; Fujimoto and Yamasoba, 2014; Benkafadar et al., 2019). The premature SAMP8 senescence causing early presbycusis was linked to altered levels of antioxidant enzymes and decreased activity of complexes I, II, and IV, which in turn lead to chronic inflammation and triggering of apoptotic cell death pathways (Menardo et al., 2012). Even before the premature onset of hearing loss, SAMP8 cochleae already displayed an early increase in ROS levels and downregulation of antioxidant enzymes (Menardo et al., 2012; Benkafadar et al., 2019). The rapidly aging SAMP8 strain may provide a useful model for studying the effects of aging on biological processes, especially regarding ARHL pathophysiology. However, the SAMP8 population exhibits a large variability in their age-related decline of auditory function. The extent of this functional variability and the underlying cellular degeneration pattern have thus far not been systematically studied.

In the present study, the auditory function of SAMP8 mice was analyzed at the age of 6 weeks (juvenile), 12 weeks (young adult), and 24 weeks (adult; Wang et al., 2020) by measuring auditory brainstem response (ABR) thresholds. We observed a bifurcation into two subgroups, i.e., in lower and upper quartiles with respect to different progressions of age-related ABR threshold increase. We investigated the variability in auditory function by contrasting these two classifications in a spatiotemporal histological analysis. For each age group and subgroup, cytocochleograms were created, which cartographically represent the number of OHCs and IHCs along the cochlear length (Viberg and Canlon, 2004; Müller et al., 2005; Carignano et al., 2019). A frequency-place map was used to correlate functional changes measured by the ABR to morphological insults in the organ of Corti (OC). Here, OHC loss observed over age could not explain threshold variability within the age groups. Therefore, cochlear cross-sections were analyzed for membrane expression of KCNQ4 (K_V_7.4) and KCNQ1 (K_V_7.1). KCNQ4 is a K^+^ channel expressed in the basal pole of mature OHCs and is responsible for their resting potential and I_K_,_n_ current (Housley and Ashmore, 1992; Santos-Sacchi, 1993; Kros et al., 1998), which is critical for OHC function (Jentsch, 2000a, b; Kharkovets et al., 2006; Gao et al., 2013; Carignano et al., 2019). And KCNQ1 is expressed in marginal cells of the SV where it mediates the slow delayed rectifier current I_K,s_ (Barhanin et al., 1996; Sanguinetti et al., 1996; Neyroud et al., 1997; Shen and Marcus, 1998) and forms an essential component in K^+^ circulation and secretion into the endolymph, generating the EP. We hypothesize that diminished levels of KCNQ4 and KCNQ1 constitute possible candidates for phenotypic change preceding OHC apoptosis, which may account for the observed auditory threshold variability in SAMP8 mice.

## Materials and Methods

### Animals and Experimental Design

Senescence-accelerated mouse prone strain 8/TaHsd mice of either sex were acquired from Envigo (Horst, Netherlands) at an age of 30 days and allowed to acclimatize for 7 days in an in-house animal facility. They were held in groups of one (only aggressive males) to five animals in a standard macrolon polycarbonate housing and were maintained on a 12-h light-dark cycle with access to food and water *ad libitum*. All animals were handled and housed according to the German (TierSchG) and European Union (directive 2010/63/EU) guidelines for the protection of animals used for experimental purposes, as reviewed and approved by the veterinary care unit of the University of Tübingen and by the regional Animal Care and Ethics Committee (Regierungspräsidium Tübingen).

The experimental design consisted of two cohorts of SAMP8 mice. In a **reference cohort** of 84 animals, ABR thresholds were collected from accumulated control groups that were divided into three age groups: juvenile (average age of 6 weeks), young adult (average age of 12 weeks), and adult (average age of 24 weeks). This cohort served as a reference for age-dependent development of ABR threshold distributions.

**FIGURE 1:**
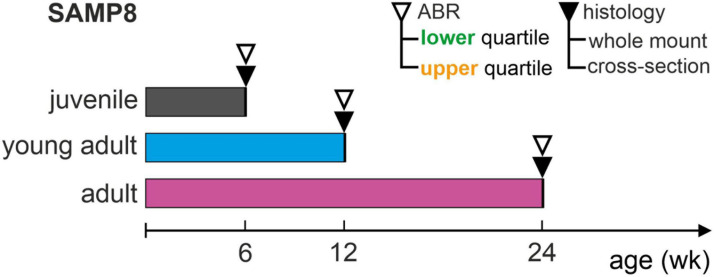
Schematic representation of the experimental design. Auditory function over age was assessed in SAMP8 mice by determining auditory brainstem response (ABR) thresholds for each age group: juvenile (6 weeks), young adult (12 weeks), and adult (24 weeks). ABR thresholds were compared with respective reference distributions from accumulated controls for each age group (reference cohort), and then allocated to the lower (generally below the 25th percentile) or upper (generally above the 75th percentile) quartiles. Following this, mice in the lower or upper quartiles were sacrificed and their cochleae extracted for histology, using one cochlea for whole-mount and the other for cross-section analysis. This study included a total of 22 mice (out of 46 measured).

In the **experimental cohort** of 46 animals, ABR thresholds were measured for each age group (juvenile, young adult, or adult) and compared to the interquartile range (between the 25th and 75th percentile) of the age-respective distribution in the reference cohort. In order to correlate functional ABR threshold distributions with significant cellular changes in a cytohistologic analysis, mice were selected based on ABR thresholds below the 25th percentile (lower quartile) and above the 75th percentile (upper quartile). Following this classification for each age group, three (to four) mice from the lower or upper quartile of the respective reference cohort were sacrificed and their cochleae extracted for histologic analysis, with one cochlea used for the generation of cytocochleograms from surface whole-mount preparations and the other for cross-sectional analysis. In this experimental cohort, the total of 46 mice only underwent ABR measurements and 22 mice met the selection criteria and were thus included for histological analysis. An overview of the experimental design is shown in Figure 1.

### Auditory Brainstem Response

Auditory brainstem response was recorded in response to pure-tones at frequencies 2.0–45.2 kHz in 2 steps per octave, with a duration of 3 ms and 1 ms rise and fall times. Stimuli were presented at levels 10–100 dB SPL in 3 dB steps in alternating condensation and rarefaction polarities. A multi-function I/O-card (National Instruments, Austin, TX, United States) was used to generate the stimulus waveforms and record the ABR signal. Acoustic stimuli were delivered in a calibrated open-field system using a dynamic loudspeaker placed lateral to the respective auricle of the animal. The sound pressure level was calibrated before each measurement block using a microphone probe (Brüel & Kjaer Types 4939 and 2670, Nærum, Denmark) placed near the entrance of the external auditory canal in line with the loudspeaker at 90° incidence. The ABR signal was recorded using a differential amplifier between silver-wire electrodes inserted subcutaneously on the vertex (positive terminal), on the mastoid of the respective ear (negative terminal) and on the back (ground), with a gain of 80 dB. Signals were filtered between 100 Hz and 5 kHz using sixth-order Butterworth low- and high-pass filters and then processed by the Audiology Lab software (Otoconsult, Frankfurt am Main, Germany) after analog-to-digital conversion at 50 kHz sampling frequency. ABR recordings were conducted in a soundproof chamber (IAC Acoustics, Niederkrüchten, Germany). ABR thresholds are defined as the sound pressure level at which a stimulus-related response was clearly identified by visual inspection of the ABR signal averaged from 128 stimulus repetitions for each polarity.

All animals were anesthetized during the ABR measurement by intraperitoneal injection of 0.05 mg/kg fentanyl (Fentadon, Dechra, Aulendorf, Germany), 0.5 mg/kg medetomidine hydrochloride (Dormilan, alfavet, Neumünster, Germany) and 2.5 mg/kg midazolam (Hameln Pharma plus GmbH, Hameln, Germany). To preserve eye moisture, an ointment (Bepanthen, Bayer AG, Leverkusen, Germany) was applied. Over the course of the measurement, electrocardiography was monitored and the animals were placed on a heating blanket to maintain their body temperature at approximately 37°C.

### Histology

After the ABR measurement and allocation to the lower or upper quartile of the respective reference cohort, pre-anesthetized mice were sacrificed by intracardial injection of 600 mg/kg pentobarbital-natrium (Narcoren, Boehringer Ingelheim, Ingelheim am Rhein, Germany) followed by decapitation. Temporal bones were subsequently removed and the cochleae isolated, with one cochlea used for whole-mount and the other for cross-section analysis.

#### Cochlear Whole-Mount Preparation

To generate cochlear whole-mount preparations, temporal bones were dissected on ice, perfused with 4% formaldehyde and decalcified in 0.2 M EDTA for 27 h at 4°C. Once decalcification was completed, dissection of the cochlear sensory epithelium, i.e., the OC, was performed under a stereo microscope (Zeiss Stemi 200-C, Oberkochen, Germany). For OC extraction, the bony labyrinth was removed, followed by a detachment of the SV and a separation of the sensory epithelium from the spiral ganglion. The OC was divided into three segments designated as apical, middle, and basal segments. The three segments for each cochlea were transferred into one well of a 48-well plate filled with 500 μl of PBS.

For fluorescence and immunofluorescence labeling, the prepared whole-mount preparations were permeabilized using 0.2% Triton X-100 in PBS for 20 min at room temperature and immersed in a blocking buffer containing 0.2% Triton X-100 in PBS and 1% normal donkey serum (NDS) for 30 min at room temperature. Whole-mount preparations were incubated with anti-Myosin VIIa (1:400, rabbit, Developmental Studies Hybridoma Bank, Iowa City, IA, United States) and anti-CtBP2 (1:200, monoclonal mouse, BD Biosciences, San Jose, CA, United States) antibodies and visualized with an Alexa 488-conjugated anti-rabbit secondary antibody (1:400, Invitrogen, Paisley, United Kingdom) and an Alexa 647-conjugated anti-mouse secondary antibody (1:400, Invitrogen). All antibodies were diluted in PBS that had been supplemented with 0.2% Triton X-100 and 0.5% NDS. For nuclear and F-actin fluorescence staining, samples were incubated for 20 min at room temperature in DAPI (1:100, Sigma-Aldrich, St. Louis, MO, United States) and Phalloidin 568 (1:400, Invitrogen) and coverslipped using FluorSave mounting medium (Calbiochem, Merck, Darmstadt, Germany). Immunolabelled whole-mount preparations divided into apical, middle, and basal segments were imaged at 10× magnification using an epifluorescence microscope (Zeiss Axioplan 2 with an ApoTome.2 unit).

#### Cochlear Cross-Sections

For generation of cochlear cross-sections, isolated cochleae were fixed by immersion in 2% paraformaldehyde and 125 mM sucrose in 100 mM PBS (pH 7.4) for 2 h. After fixation, cochleae were decalcified for 45 min in RDO rapid decalcifier (Apex Engineering Products Corporation, Aurora, IL, United States). Cochleae were stored overnight at 4°C in 25% Sucrose-Hank’s solution and, on the following day, embedded in Tissue-tek (Sakura Finetek, AV, Netherlands). After embedding, cochleae were cryosectioned at 12 μm and mounted on SuperFrost Plus microscope slides (Thermo Fisher Scientific, Waltham, MA, United States) before storage at −20°C.

For fluorescence and immunofluorescence labeling of KCNQ4, microscope slides with mounted cochlear sections were permeabilized in 0.1% Triton-X 100 in PBS for 10 min and incubated for 30 min in a humidified chamber in blocking buffer containing 1% BSA in PBS. Cochlear sections were stained with antibodies against prestin (1:3000, rabbit, Squarix, Berlin, Germany) and KCNQ4 (1:50, mouse, StressMarq, Victoria, BC, Canada). For labeling of KCNQ1, microscope slides with mounted cochlear sections were permeabilized in 0.5% Triton-X 100 in PBS for 10 min and incubated for 30 min in blocking solution containing 4% normal goat serum (NGS) in PBS. Cochlear sections were stained with antibodies against KCNQ1 (1:200, rabbit, Alomone Labs, Jerusalem, Israel) and KCNJ10 (K_ir_4.1, 1:50, mouse, Sigma-Aldrich). To detect primary antibodies, Cy3 anti-rabbit (1:1500, Jackson Immuno Research Laboratories, West Grove, PA, United States) and Alexa477-conjugated anti-mouse secondary antibodies (1:500, Invitrogen) were used. Antibodies against prestin, KCNQ4, and corresponding secondary antibodies were diluted in 0.5% BSA in PBS, whereas antibodies against KCNQ1, KCNJ10, and corresponding secondary antibodies were diluted in a reaction buffer containing 0.1% Triton-X 100 in PBS, 2% NaCl, and NGS. For nuclear staining, Vectashield mounting medium with DAPI (Vector Laboratories, Burlingame, CA, United States) was used to mount microscope slides with cochlear sections.

Sections were viewed using an Olympus BX61 microscope (Olympus, Hamburg, Germany) equipped with epifluorescence illumination and analyzed with cellSens Dimension software (OSIS GmbH, Münster, Germany). To increase spatial resolution, slices were imaged over a distance of 15 μm within an image-stack along the *z*-axis (z-stack) followed by three-dimensional deconvolution using cellSens Dimension’s built-in algorithm.

### Data Analysis

#### Cytocochleogram

Images of OC whole-mount preparations were analyzed using the ImageJ software (NIH, Bethesda, MD, United States) and cell counting was carried out manually by assigning different markers stored with their coordinates using the plug-in “Cellcounter.” For each OC whole-mount segment (apical, middle, or basal), the length was measured along the clearly defined junction between the outer pillar cell and the first row of OHCs. The line was traced along the longitudinal axis of the OC across all three segments to determine the full spiral length. OHCs and IHCs were counted as present if co-staining of three cellular markers consisting of the cellular nucleus (DAPI), the stereocilia bundle (phalloidin), and the cellular cytoplasm (Myosin VIIa) were evident. Otherwise, if one of these markers was absent, hair cells were counted as missing. Each marker (present or missing OHCs and IHCs) was allocated to the nearest point on the spiral of the OC by calculating the minimum Euclidean distance. The total OC spiral length was determined by concatenating the three whole-mount segments. Then, the length was normalized to 100% and the count of present or missing OHCs and IHCs, respectively, was calculated as a function of the relative distance from the apex subdivided into 5% bins. Thereby, the extent and pattern of lost cells and the remaining intact cells along the cochlear length were mapped as a “cytocochleogram” (Engström et al., 1966; Viberg and Canlon, 2004; Müller et al., 2005; Carignano et al., 2019).

#### OHC and SV Phenotypes

For each age group and for either KCNQ4 or KCNQ1 analysis, pairs of cochlear cross-sections from two mice in the lower and upper quartiles, respectively, were stained on the same day using the same corresponding reagent solutions (see section “Histology”). Each pair was then imaged together using the same confocal acquisition parameters and exposure times. The presence of an OHC was verified if both the cell nucleus as well as the OHC-specific motor protein prestin were labeled, then KCNQ4 membrane expression at the basal pole of at least three OHCs was localized. The expression of KCNJ10 in the intermediate layer of the SV was used to delimit the marginal layer with KCNQ1 expression. Thereafter, a 100× oil immersion lens was used to image the OC and the SV, respectively, at an apical, middle, and midbasal position.

Quantitative analysis of KCNQ4 and KCNQ1 was performed in fluorescence images of cochlear cross-sections using the ImageJ software. For each magnified image, the background was reduced using the “rolling ball” algorithm (built-in ImageJ tool) with a radius of six pixels. The regions of interest (ROIs) used for quantification were delimited by circles with a diameter of 100 pixels around the basal pole of OHCs for the KCNQ4 staining, and by rectangles with a fixed width of 100 pixels along the marginal layer of the SV for the KCNQ1 staining. Subsequently, delimited ROIs were inserted onto a black background and the corresponding color channel (green for KCNQ4 and red for KCNQ1) was split and converted to an 8-bit grayscale image with a threshold of 75 pixel intensity to minimize contributions from background staining. Subsequently, pixel intensity was integrated over the processed images to obtain the area under the curve (AUC), then normalized by the number of OHCs to obtain a mean KCNQ4 intensity per OHC, or by the SV length to obtain a mean KCNQ1 intensity per SV width.

#### Statistics

Data are presented as mean with standard deviation (SD), or with standard error of the mean (SEM) for small sample sizes (*n* = 3 or 4) as an estimate of the deviation of the sample mean from the population mean. Data distributions were tested for normality by the Shapiro–Wilk test. Pairwise differences of means were compared for statistical significance using the Mann–Whitney-*U* for two samples or the Kruskal–Wallis test for more than two samples. Least-squares linear regressions were assessed by the coefficient of determination *R*^2^. And linear correlations were determined by Pearson’s correlation coefficient *r*. A *p*-value of less than 0.05 or less than 0.001 was considered statistically significant or highly significant, respectively. Statistical analysis was performed using IBM SPSS Statistics 27 (IBM Corporation, Armonk, NY, United States).

## Results

### SAMP8 Mice Develop Progressive Age-Related ABR Threshold Increase

**FIGURE 2:**
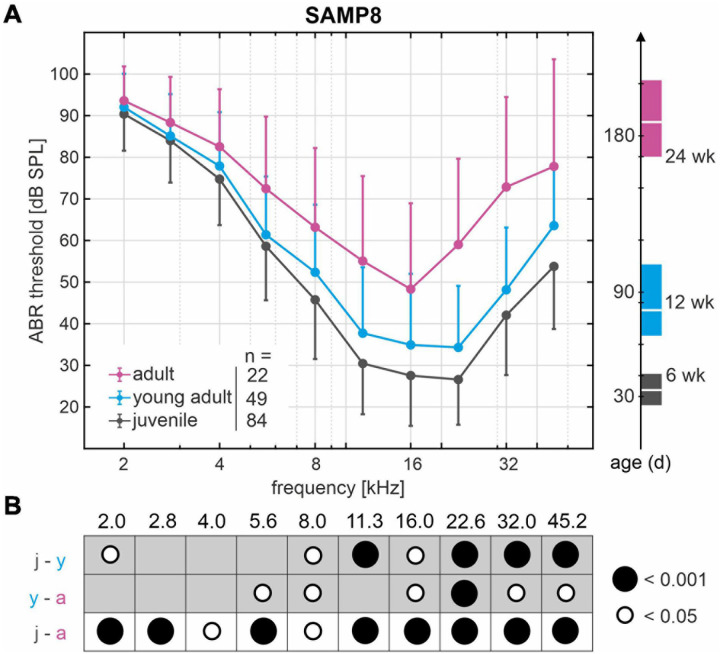
Decline of hearing acuity over age in SAMP8 mice as assessed by auditory brainstem response (ABR) thresholds. **(A)** Mean and standard deviation of ABR thresholds from accumulated control groups (reference cohort) divided into three age groups: juvenile (black, *n* = 84), young adult (blue, *n* = 49), and adult (pink, *n* = 22). The right graph shows mean age and age range in days for each group: juvenile (black, mean 34 days, range 25–45 days), young adult (blue, mean 80 days, range 65–105 days), and adult (pink, mean 188 days, range 170–210 days). **(B)** Graphical representation of significant differences (white and black circles represent *p* < 0.05 and *p* < 0.001, respectively) from pairwise comparisons of ABR thresholds between age groups (j, juvenile; y, young adult; a, adult) for each frequency in kHz.

ABR in SAMP8 mice were assessed over age by pure-tone stimulus-evoked ABR thresholds from accumulated control groups (reference cohort) that were divided into three age groups: juvenile (*n* = 84, mean 34 ± 6 days, range 25–45 days), young adult (*n* = 49, mean 80 ± 11 days, range 65–105 days), and adult (*n* = 22, mean 188 ± 14 days, range 170–210 days). Mean and SD of ABR thresholds for frequencies 2.0–45.2 kHz as well as corresponding mean age and age range are shown for each age group in Figure 2A. Comparison of the ABR thresholds across frequencies between age groups revealed a progressive increase in hearing loss starting in the high-frequency range (>8 kHz) between juvenile and young adult stages and further progressing toward the low-frequency range (≤8 kHz) in the adult stages. The significant differences between age groups from pairwise comparisons of ABR thresholds are indicated graphically in Figure 2B for each frequency, with young adult SAMP8 mice already exhibiting a significant increase of hearing thresholds for high frequencies (>8 kHz) compared with juvenile mice (*p* < 0.05). In adult mice, ABR thresholds were further elevated across all frequencies when compared with young adult mice, reaching statistical significance at 5.6 and 8 kHz (*p* < 0.05), and at 16–45.2 kHz (*p* < 0.05). When compared with juvenile mice, significantly elevated ABR thresholds were found in adult animals for all frequencies (*p* < 0.05). The mean increase in ABR threshold between juvenile and adult SAMP8 mice was 9.3 dB for the low-frequency range (≤8 kHz) and 26.5 dB for the high-frequency range (>8 kHz). SAMP8 mice thus show an early progressive, age-related increase in ABR thresholds, starting primarily in the high-frequency range and later extending to all frequencies at an adult age.

### SAMP8 Mice Exhibit Variability in ABR Threshold Progression

**FIGURE 3:**
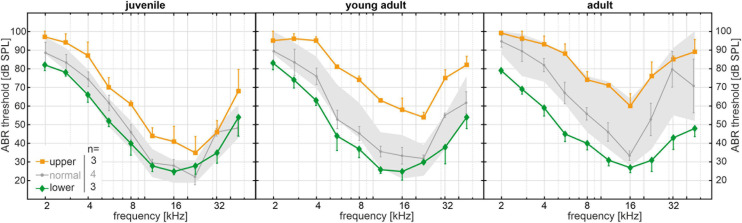
Long-term SAMP8 monitoring cohort (*n* = 10) with auditory brainstem response (ABR) thresholds measured at juvenile (34 days), young adult (80 days), and adult age (195 days). ABR thresholds were compared with the interquartile range of the reference cohort for each age group (gray area, see Figure 2) and divided into subgroups in the lower quartile (green diamonds, *n* = 3), in the normal range (gray circles, *n* = 4), and in the upper quartile (orange squares, *n* = 3) based on their progression of age-related threshold increase over age. For each age group and subgroup, mean and standard error of the mean ABR thresholds are shown.

Apart from the significantly progressive, age-related ABR threshold increase found in the SAMP8 reference cohort, the threshold variability for each age group–as represented by the SD–also appears to increase as a function of age (Figure 2A). In line with this observation, the mean SD of ABR thresholds over all frequencies in adult mice (18 ± 5 dB SPL) was significantly higher than in juvenile mice (12 ± 2 dB SPL, *p* < 0.05). We hypothesize a bifurcation into subgroups with slow or fast progression of ABR threshold increase over age, which presumably entails the significant increase in threshold variability between age groups. To identify the lower and upper ends of the threshold distribution within the SAMP8 subgroups, we compared the ABR threshold of a **monitoring cohort** of SAMP8 mice (*n* = 10, five males and five females) at juvenile (34 days), young adult (80 days), and adult age (195 days) with the interquartile range of the reference cohort for each age group. The monitoring cohort was then divided into subgroups (lower, normal, or upper) based on their progression of age-related ABR threshold increase. For each classification and age group, mean and SEM of ABR thresholds (one ear for each mouse) are shown in Figure 3. Mice in the lower quartile (lower, *n* = 3) showed a relatively slow progression of ABR threshold increase over age, tending toward a resistance against age-related decline. By contrast, mice in the upper quartile (upper, *n* = 3) showed a relatively fast progression. Finally, mice in the normal-hearing subgroup (normal, *n* = 4) adhered to the progression found in the reference cohort. No correlation was found between these classifications and the sex of the mice, and no relevant asymmetries were found between ears for each mouse. Note that at juvenile age, SAMP8 mice allocated to the upper quartile already showed elevated ABR thresholds for all frequencies when compared with either normal-hearing mice (mean shift 11 ± 5 dB) or mice in the lower quartile (mean shift 16 ± 4 dB). By contrast, normal-hearing mice and mice allocated to the lower quartile were initially comparable at juvenile age (mean shift 4 ± 6 dB), then progressively diverged up to the adult age (mean shift 18 ± 8 dB). In summary, the monitoring SAMP8 cohort confirmed an age-dependent bifurcation into subgroups with different progressions of age-related ABR threshold increase. In order to address related morphological alterations, a spatiotemporal histological analysis was performed for the different age groups.

### ABR Threshold Loss of SAMP8 Mice Partly Independent of Cochlear Hair Cell Loss

**FIGURE 4:**
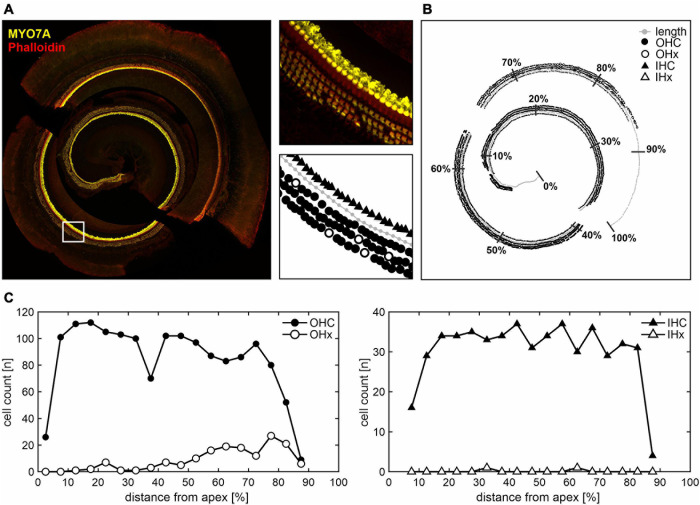
Hair cell count along the cochlear spiral length in whole-mount histological analysis. **(A)** Illustrative example of apical, middle, and basal cochlear segments stained with MYO7A for hair cells and phalloidin for stereocilia (left panel). In the white-square delimited area, different marker assignments are illustrated (right panels): cochlear length axis (gray circles), present or missing outer hair cells (OHC as black circles, or OHx as white circles, respectively), and present or missing inner hair cells (IHC as black triangles, or IHx as white triangles, respectively). **(B)** The corresponding overview is shown with the cochlear length normalized to 100% distance from apex and subdivided into 10% bins. Due to preparation difficulties, some whole-mount segments were only partially intact, e.g., in the 90–100% range. These were not included in the analysis, i.e., no hair cell markers were assigned, whereas the length axis was still used to approximate the total cochlear spiral length. **(C)** Present or missing OHC (left panel) and IHC (right panel) counts are shown as a function of the relative distance from apex in 5% bins, with the cell count plotted in the middle of the respective bin. Note that entire bins without valid hair cell markers were excluded, while bins with only partially valid markers due to preparation difficulties were included, but showed generally lower cell counts.

Outer hair cell survival were analyzed performing cytocochleograms obtained from OC whole-mount preparations of juvenile, young adult, and adult aged SAMP8 mice. According to the functional ABR threshold classification, SAMP8 mice were selected from the lower and upper quartiles as described above for each of the three age groups. The two functionally different groups were chosen to gain insight into potential cellular correlates of the observed diverging threshold losses. An illustrative example of analyzing OC whole-mount segments (apical, middle, and basal) is shown in Figure 4A. Present or missing OHCs and IHCs were counted and allocated to bins along the normalized spiral length of the OC. The mean length of the OC was 5.33 ± 0.36 mm (*n* = 19). In some cases, whole-mount segments were only partially intact due to preparation artifacts, e.g., in the 90–100% range of this illustrative example (Figure 4B). These were not included in the analysis while the length axis was maintained to allocate the hair cell count to the correct position along the total length of the OC. Cell counts of present or missing OHCs and IHCs as a function of the relative distance from apex in 5% bins are demonstrated in Figure 4C. Note that entire bins without valid hair cell markers were excluded from the analysis. However, bins with only partially valid markers due to preparation difficulties were included but showed generally lower cell counts.

**FIGURE 5:**
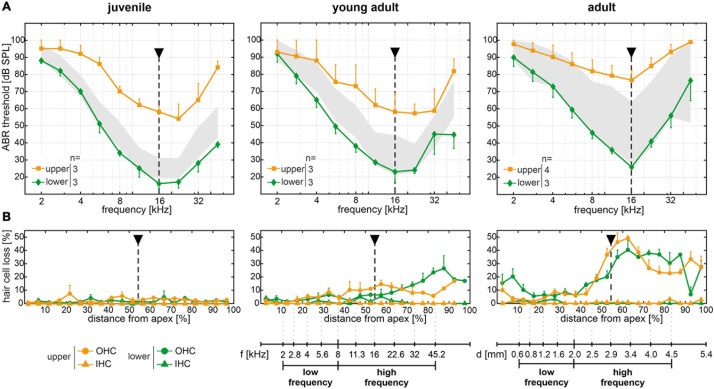
Spatiotemporal analysis of cochlear hair cell loss over age of the experimental cohort. **(A)** Mean and SEM of auditory brainstem response (ABR) thresholds of ears corresponding to the whole-mount analysis for each mouse, for mice allocated to the lower (green diamonds) and upper (orange squares) quartiles in each age group (juvenile, young adult, and adult). The interquartile range of ABR thresholds of the reference cohort for each age group is shown for comparison (gray area, see Figure 2). **(B)** Cytocochleograms showing mean and SEM of outer and inner hair cell loss (OHC as circles and IHC as triangles, respectively) as a function of relative distance from apex, for mice with ABR thresholds allocated to the lower (green) and upper (orange) quartiles, respectively, in each age group. A mouse place-frequency map (Wang et al., 2002; Viberg and Canlon, 2004; Müller et al., 2005) was used to divide cytocochleograms into low- (≤8 kHz) and high-frequency (>8 kHz) ranges. For reference, frequency (f) and cochlear spiral length (d) axes are depicted with respect to the relative distance from apex. The dashed line highlights ABR thresholds and corresponding OHC and IHC loss at 16 kHz, for which the largest mean threshold difference was found in all age groups.

In the following, cytocochleograms are presented as the ratio between missing hair cells and the sum of present and missing hair cells, i.e., percentage of hair cell loss, in each bin. Thereby, the limitation of only partially valid markers in a given bin was overcome and the comparison between relative OHC and IHC loss was facilitated. The mean age at the ABR measurement followed by cochlear extraction for mice in either quartile was 38 ± 4 days (juvenile), 97 ± 15 days (young adult), and 184 ± 16 days (adult). At juvenile age, mice in the upper quartile (*n* = 3) had elevated ABR thresholds across all frequencies compared with same-aged mice in the lower quartile (*n* = 3). The most pronounced difference was seen at 16 kHz (Figure 5A, dashed line). At this frequency, juvenile mice in the lower quartile had a mean threshold of 16 ± 3 dB SPL, whereas mice in the upper quartile had a mean threshold of 58 ± 3 dB SPL, i.e., the mean threshold difference at 16 kHz was 42 dB. At young adult age, the mean threshold difference was 35 dB between the respective mice in the lower (*n* = 3) and upper (*n* = 3) quartiles. And at adult age, the difference was 51 dB between the respective mice in the lower (*n* = 3) and upper (*n* = 4) quartiles. Note that at adult age, mice in the upper quartile had profound hearing loss with a mean threshold of 77 ± 6 dB SPL at 16 kHz. By contrast, the same-aged mice in the lower quartile had a mean threshold of 26 ± 1 dB SPL, which lies within the interquartile range of the reference cohort at juvenile age, i.e., they had a decelerated progression of ABR threshold increase over age. This is in line with the observations made in the SAMP8 monitoring cohort with continuous ABR measurements (see Figure 3).

Cytocochleograms corresponding to mice of the experimental cohort with ABR thresholds allocated to the lower or upper quartiles over age are plotted in Figure 5B. IHC loss remained below 5% for mice in either quartile, for all age groups. Surprisingly, no OHC loss was observed in both the lower and upper quartiles at all frequencies of the juvenile age group despite a mean threshold difference of 42 dB at 16 kHz. In young adult mice, OHC loss was found starting at an approximately 40% distance from apex, which corresponds to about 9 kHz as estimated by a mouse place-frequency map (Wang et al., 2002; Viberg and Canlon, 2004; Müller et al., 2005), and progressed toward the basal region. In adult mice, OHC loss further increased in the same regions. However, no consistent differences were found between respective mice in the lower and upper quartiles. At young adult age, for example, the distance from apex corresponding to 16 kHz (Figure 5B, dashed line) had a mean OHC loss of 7 ± 2% in the lower (*n* = 3) and 12 ± 2% in the upper (*n* = 3) quartiles, which is in contrast to a mean threshold difference of 35 dB between these groups. At adult age, the mean OHC loss was 21 ± 8% in the lower (*n* = 3) and 38 ± 9% in the upper (*n* = 4) quartiles. This presents a relatively larger difference between the two groups and functionally compares to a mean threshold difference of 51 dB. At more basal sites (70–90% distance from apex), young adult and adult mice in the respective upper quartiles showed less OHC loss than those in the respective lower quartiles, which appears contradictory to the ABR threshold difference at the same frequency locations between mice in the upper and lower quartiles.

**FIGURE 6:**
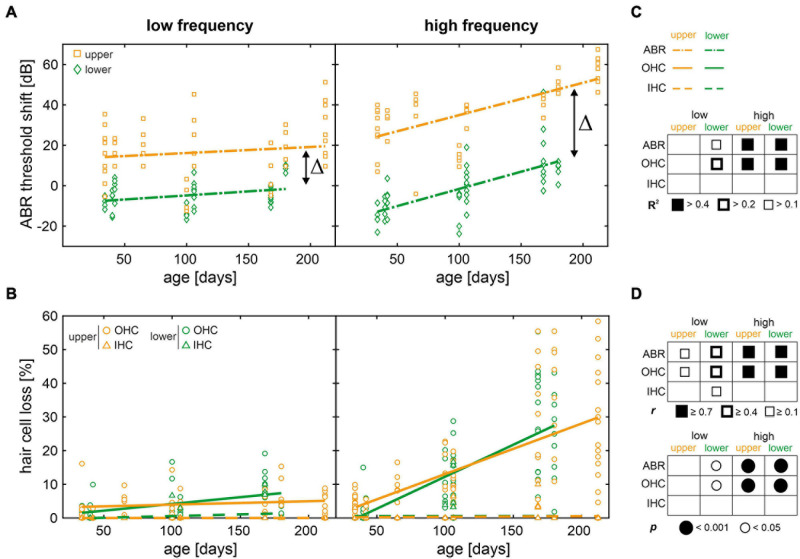
Scatterplots of auditory brainstem response (ABR) threshold shifts and hair cell loss as a function of age with regression and correlation analysis. **(A)** Individual ABR threshold shifts over age for mice in the lower (green diamonds) and upper (orange squares) quartiles, in the low- (≤8 kHz) and high-frequency (>8 kHz) range, respectively. ABR threshold shifts were calculated for each mouse by subtracting the mean threshold of the reference cohort at juvenile age (see Figure 2) from each given threshold, at each given frequency. Fitted linear regression models (dash-dotted lines) are shown for mice in the lower (green) and upper (orange) quartiles, respectively. Δ indicates the difference of intercepts between models for mice in the lower and upper quartiles, for each frequency range. **(B)** Individual outer and inner hair cell loss (OHC as circles and IHC as triangles, respectively) over age for mice in the lower (green) and upper (orange) quartiles, in the low- and high-frequency range, respectively. Fitted linear regression models for OHC loss (solid lines) and for IHC loss (dashed lines) are shown for mice in the lower (green) and upper (orange) quartiles, respectively. **(C)** Graphical representation of the coefficient of determination (*R*^2^) ranges of the linear regression models for each measure and for each condition. **(D)** Graphical representation of correlation coefficients (Pearson’s *r*, upper table) and corresponding significance values (*p*, lower table) for each measure and for each condition.

To further quantify ABR threshold shifts and OHC loss over age, a mouse place-frequency map (Viberg and Canlon, 2004) was used to convert the relative distance from apex to corresponding frequencies. The cytocochleograms were then divided into low- (≤8 kHz) and high-frequency (>8 kHz) ranges in accordance with the progressive OHC loss observed in the middle and basal regions (>40% distance from apex) in contrast to the apical region (see Figure 5B). As an equivalent to relative hair cell loss, ABR threshold shifts were calculated for each mouse by subtracting the mean threshold of the reference cohort at juvenile age (see Figure 2) from each given threshold, at each given frequency. Thereby, a relative ABR threshold shift from the population mean with the juvenile age as reference could be compared between mice allocated to the lower or upper quartiles over age. Within each frequency range (low or high frequency) and for each classification (lower or upper quartile), scatterplots as a function of absolute age in days and corresponding linear regression models are shown for ABR threshold shifts in Figure 6A and for OHC or IHC losses in Figure 6B. The coefficient of determination (*R*^2^) of the linear regression models is indicated graphically in Figure 6C. And corresponding correlation coefficients (Pearson’s *r*, upper table) and their significance value (*p*, lower table) are indicated graphically in Figure 6D.

In the low-frequency range, only mice allocated to the lower quartile showed relevant linear regressions and had moderate positive correlations between age and ABR threshold shift (*r* = 0.42, *p* = 0.005) and OHC loss (*r* = 0.44, *p* = 0.002, see Figures 6C,D). In the high-frequency range, by contrast, ABR threshold shift as well as OHC loss were well-described by linear regression (*R*^2^ > 0.4) and had strong positive correlations with age (*r* ≥ 0.7, *p* < 0.001) for either quartile. By contrast, IHC loss was not significant over age as shown above (see Figure 5B). In other words, the increase of ABR threshold over age was observed in the high-frequency range independent of quartile allocation, while thresholds at lower frequencies remained largely unchanged. These observations are in line with age-related OHC loss, showing little loss in the low-frequency range, but a significant loss in the high-frequency range over age. Although these results confirm OHC loss as one predictor of ABR threshold increase over age in SAMP8 mice, the observed threshold variability cannot be explained by OHC loss alone. While OHC loss is comparable between mice allocated to the lower and upper quartiles (see Figure 6B), this is in stark contrast to the ABR threshold difference in the high-frequency range between these groups (Δ = 38 dB, see Figure 6A) which persists over age. Furthermore, the hypothesis that mice in the lower and upper quartiles exhibit slow and fast progression of age-related ABR threshold increase, respectively, is not supported by comparable slopes of the linear regression models in the high-frequency range, with a threshold increase of 8.5 dB per 50 days in the lower and 8.0 dB per 50 days in the upper quartile. This implies that lower and upper quartiles have different time points of hearing loss onset, but progress with the same rate thereafter. Despite the largely unchanged ABR thresholds over age in the low-frequency range, a persisting threshold difference between mice in the lower and upper quartiles was observed (Δ = 22 dB). Based on these observations, it is suggested that OHC loss is a secondary predictor of ABR threshold increase over age in either quartile given that functional impairment, as measured by increase in ABR thresholds, preceded OHC loss in both lower and upper quartiles.

### OHC and SV Phenotypic Differences of SAMP8 Mice Relate to ABR Threshold Variability

**FIGURE 7:**
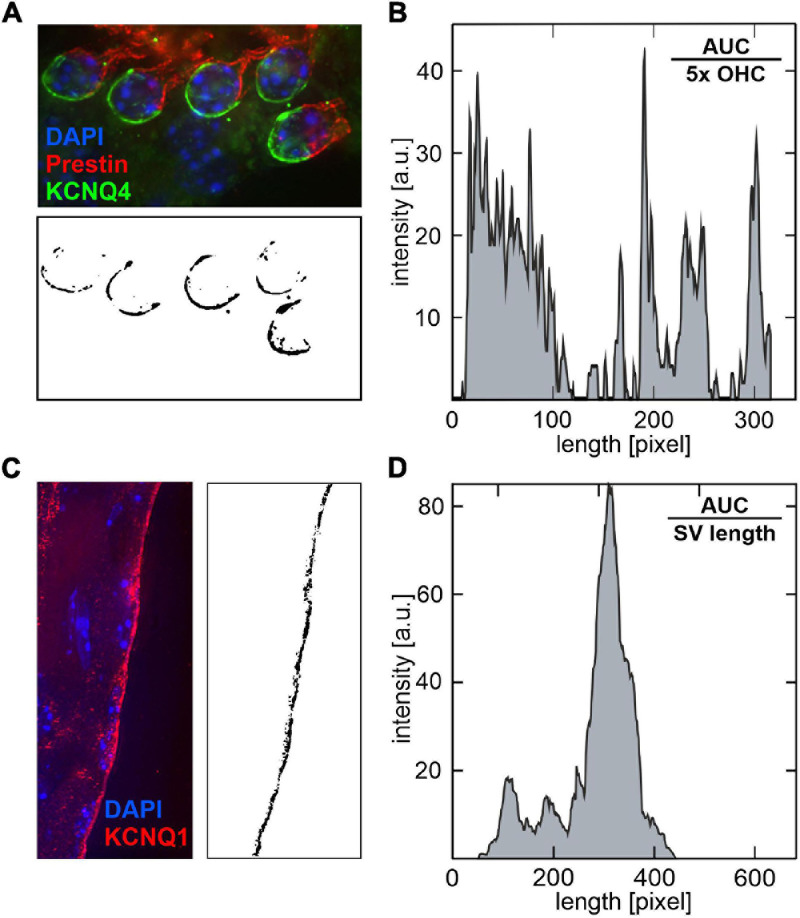
Quantitative analysis of KCNQ4 and KCNQ1 pixel intensity profiles in cochlear cross-section fluorescence images. **(A)** Illustrative example of a cochlear cross-section stained with KCNQ4 (green), prestin (red) for the outer hair cell (OHC) motor protein, and DAPI (blue) for cell nuclei (upper panel). For each image, the regions of interest (ROIs) were delimited by circles around the basal pole of OHCs. The green channel (KCNQ4) was split and converted to an 8-bit grayscale image (lower panel). **(B)** The corresponding pixel intensity profile is shown. The pixel intensity was integrated over the processed image to obtain the area under the curve (AUC) and normalized by the number of OHCs, here 5×, to calculate a mean KCNQ4 intensity per OHC. **(C)** Illustrative example of a cochlear cross-section stained with KCNQ1 (red) in the stria vascularis (SV), and DAPI (blue, left panel). ROIs were delimited by rectangles with a fixed width along the marginal layer of the SV. The red channel (KCNQ1) was split and converted to an 8-bit grayscale image (right panel). **(D)** The corresponding pixel intensity profile is shown. The AUC was normalized by the SV length along the *y*-axis to calculate a mean KCNQ1 intensity per SV width.

Given that OHC loss over age could not be linked to the observed variability of ABR thresholds between SAMP8 mice in the lower and upper quartiles, we hypothesized that potential sensitive phenotypic variances preceding OHC loss may be linked to altered levels of KCNQ4 (K_V_7.4) membrane expression in OHCs or to altered levels of KCNQ1 (K_V_7.1) in the SV. For each age group, three (juvenile) to four (young adult and adult) pairs of cochlear cross-sections of mice in the lower and upper quartiles, respectively, were analyzed for KCNQ4 (Supplementary Figures 1–3) and KCNQ1 (Supplementary Figures 4–6) membrane expression. Quantitative analysis of KCNQ4 and KCNQ1 membrane expression was carried out by processing fluorescence images of cochlear cross-sections to reduce background and detect ROIs, then split the corresponding color channel, and evaluate the pixel intensity in 8-bit grayscale images (Figures 7A,C). The pixel intensity profiles for KCNQ4 (Figure 7B) and KCNQ1 (Figure 7D) were then integrated to obtain the AUC, then normalized by the number of OHCs to obtain a mean KCNQ4 intensity per OHC, or by the SV length to obtain a mean KCNQ1 intensity per SV width.

**FIGURE 8:**
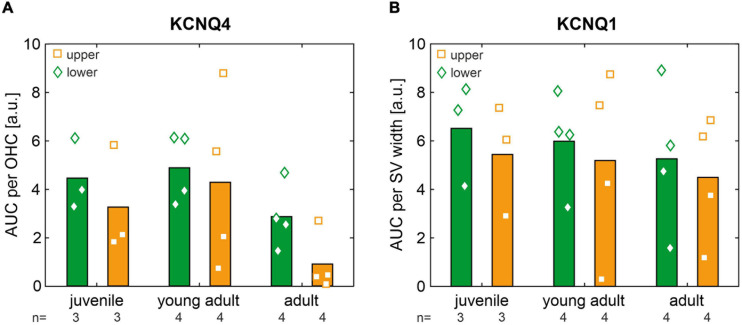
**(A)** Area under the curve (AUC) of KCNQ4 immunofluorescence normalized per outer hair cell (OHC) and averaged from cochlear cross-sections in the apical, middle, and midbasal turns for mice allocated to the lower (green diamonds) and upper (orange squares) quartiles in each age group (juvenile, young adult, and adult). Grand means are shown as bar graphs. **(B)** Same as panel **(A)** for the AUC of KCNQ1 immunofluorescence normalized per stria vascularis (SV) width.

For each age group, normalized AUCs of KCNQ4 and KCNQ1 that were averaged from cochlear cross-sections in the apical, middle, and midbasal turns, for mice allocated to the lower and upper quartiles, respectively, are shown in Figure 8. Across age groups, KCNQ4 expression (Figure 8A) was generally higher in mice allocated to the lower quartiles than in mice allocated to the upper quartiles, with the ratio of mean KCNQ4 expression in the lower quartile to the upper quartile ranging from 1.14 to 3.16. However, no significant differences were found between these quartiles for each age group. This trend was similarly observed for KCNQ1 expression (Figure 8B), with the ratio of mean KCNQ1 expression in the lower quartile to the upper quartiles ranging from 1.15 to 1.98, but no significant differences were found. Still, the data suggest that KCNQ4 and KCNQ1 membrane expressions averaged over three cochlear turns were generally reduced in the age-matched mice allocated to the upper quartiles, which is in concordance with the higher ABR thresholds found in the low- and high-frequency ranges (see Figure 6A).

**FIGURE 9:**
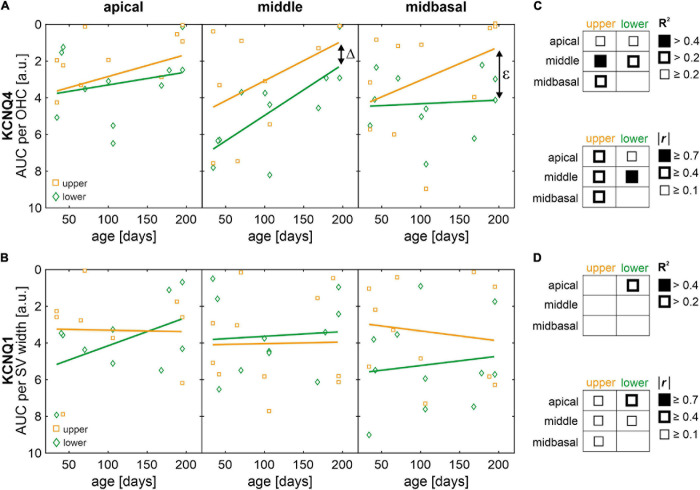
Scatterplots of normalized areas under the curve (AUC) of KCNQ4 and KCNQ1 immunofluorescence as a function of age with regression and correlation analysis. **(A)** AUC of KCNQ4 immunofluorescence per outer hair cell (OHC) over age for mice in the lower (green diamonds) and upper (orange squares) quartiles, for the apical, middle, and midbasal turns, respectively. Fitted linear regression models (solid lines) are shown for mice in the lower (green) and upper (orange) quartiles, respectively. Δ indicates the difference of intercepts between models for mice in the lower and upper quartiles and ε indicates the increase in difference over age between the lower and upper quartiles. **(B)** AUC of KCNQ1 immunofluorescence per stria vascularis (SV) width over age for mice in the lower (green diamonds) and upper (orange squares) quartiles, for the apical, middle, and midbasal turns, respectively. Fitted linear regression models (solid lines) are shown for mice in the lower (green) and upper (orange) quartiles, respectively. **(C,D)** Graphical representations of the coefficient of determination (*R*^2^, upper tables) ranges of the linear regression models and the modulus correlation coefficients (Pearson’s *r*, lower tables) for KCNQ4 **(C)** and KCNQ1 **(D)**. Note that none of the correlation coefficients was significantly different from zero.

Scatterplots as a function of absolute age in days and corresponding linear regression models for normalized AUCs of KCNQ4 and KCNQ1 are shown in Figures 9A,B, respectively, for each quartile (lower or upper) and for each cochlear turn (apical, middle, or midbasal). The coefficients of determination (*R*^2^) of the linear regression models and corresponding correlation coefficients (Pearson’s *r*) are indicated graphically for KCNQ4 and KCNQ1 in Figures 9C,D, respectively. KCNQ4 expression in OHCs showed relevant linear regressions in the middle turn with negative correlations between KCNQ4 expression and age in mice allocated to the lower (*r* = −0.71, *p* = 0.014) and upper quartiles, respectively (*r* = −0.39, *p* > 0.05, Figure 9A, middle). The persistent difference (Δ) between the lower and upper quartiles over age is congruent with the ABR threshold difference observed between these groups which also persists over age (see Figure 6A). Another relevant linear regression was found in the midbasal turn with a negative correlation between KCNQ4 expression and age in mice allocated to the upper quartile (*r* = −0.55, *p* = 0.078), while the expression in the lower quartile did not correlate with age (Figure 9A, midbasal). The apparent increase (ε) in expression difference between the lower and upper quartiles in the midbasal turn and the aforementioned, persistent difference (Δ) in the middle turn coincide with the larger ABR threshold difference observed in the high-frequency region (compare Figures 6A, 9A). With regards to KCNQ1 expression in the SV, a relevant linear regression was solely found in the apical turn for mice allocated to the lower quartile with a moderate negative correlation between KCNQ1 expression and age (*r* = −0.43, *p* > 0.05, Figure 9B, apical). Although mice allocated to either quartile generally showed weak correlations between KCNQ1 expression and age, a persistent difference over age may be interpreted between the lower and upper quartiles in the midbasal turn (Figure 9B, midbasal). Based on these observations, it is suggested that altered OHC and SV phenotypes constitute potential predictors of the observed ABR threshold differences in SAMP8 mice, feasibly preceding OHC loss over age.

## Discussion

In the present study, we analyzed the auditory thresholds and sensory hair cell loss of SAMP8 mice as a function of age. Apart from the early progressive, age-related decline in hearing acuity, we observed a significant increase in threshold variability over age. We hypothesized that SAMP8 mice exhibit different progressions of age-related ABR threshold increase. In line with this hypothesis, we found that a monitoring cohort of SAMP8 presented a bifurcation into two subgroups, i.e., in lower and upper quartiles, with respect to ABR threshold distribution. Surprisingly, in the experimental cohort, the large variability of threshold loss between these quartiles did not conclusively correlate to a cellular loss of OHCs. In fact, functional loss as measured by ABR threshold increase was found to precede OHC loss. In search for sensitive phenotypic markers of this age-related functional loss, we hypothesized that diminished expression levels of KCNQ4 (K_V_7.4), a K^+^ channel crucial for OHC function and survival, and KCNQ1 (K_V_7.1), an essential channel for K^+^ circulation and secretion into the endolymph generating the EP, constitute possible candidates for phenotypic alteration preceding OHC loss and possibly accounting for the observed threshold variability. KCNQ4 and KCNQ1 membrane expressions were found to be decreased in mice with ABR thresholds allocated to the upper quartile compared with those in the lower quartile, with partly persistent or increasing differences between these quartiles and weak negative correlations between membrane expression and age. We thus propose that diminished expression levels of KCNQ4 in OHCs and KCNQ1 in the SV may be underlying causes of the variability in auditory threshold progression of SAMP8 mice. In the following sections, we discuss the implications of these results for the SAMP8 mouse model of ARHL, the ABR threshold increase in the context of OHC or IHC loss, i.e., sensory presbycusis, and KCNQ4 and KCNQ1 expressions as well as other potential predictors affected by accumulated metabolic challenges over age that need to be investigated as possible underlying causes of ARHL preceding irreversible OHC loss in the SAMP8 mouse model.

### SAMP8 Mouse Model of ARHL

The SAMP8 strain is commonly used as a model to study several disorders observed in aging, such as senile amyloidosis, osteoporosis, cataracts, and brain atrophies (Akiguchi et al., 2017; Karuppagounder et al., 2017; Folch et al., 2018; Grinan-Ferre et al., 2018). Previously, SAMP8 mice were used to investigate premature cochlear aging and as a model of ARHL with an early progressive, age-related decline in hearing acuity (Menardo et al., 2012; Marie et al., 2017). In the present study, we confirmed these findings with our SAMP8 reference cohort showing significant ABR threshold increases from juvenile to young adult and adult age (see Figure 2). This age-related increase in ABR thresholds started in the high-frequency range and later extended to all frequencies, which closely mimics the typical audiometric progression in human ARHL, characterized by a greater deterioration of audiometric thresholds over age at high frequencies than at low frequencies (Gates and Mills, 2005; Gordon-Salant, 2005; Wu et al., 2020).

In addition to the significantly progressive age-related ABR threshold increase found in the SAMP8 population, we observed an age-dependent increase in threshold variability for each age group. The continuous ABR measurement in the monitoring cohort presented a bifurcation in thresholds showing a highly variable progression of ARHL in the SAMP8 model (see Figure 3). Previously, Benkafadar et al. (2019) compared the progression of ABR threshold increase over age between SAMP8 and the senescence-accelerated mouse resistant 1 (SAMR1) strain, which was typically used as a SAMP8 control representing normal senescence (Takeda et al., 1981). Benkafadar et al. (2019) confirmed that both strains develop ARHL after 1 month of age, but threshold progression was shown to be faster in the SAMP8 strain with a threshold increase over the frequencies 2–32 kHz of 5.0 dB/month versus 2.8 dB/month in the SAMR1. Similar progressions have also been previously reported for the compound action potential (CAP) evoked by tone bursts at 20 kHz (Menardo et al., 2012). For our SAMP8 reference cohort, we estimated a threshold increase of 3.6 dB/month over all frequencies, which seems to fall in between the progressions previously reported for the SAMP8 and SAMR1 strains.

Furthermore, individual data reported by Benkafadar et al. (2019) for either strain also presented a large ABR threshold variability as observed in the present study. Already at 1 month of age, they reported a mean ABR threshold range over all frequencies of approximately 34 dB (*n* = 14), and at 6 months of age, the range increased up to 59 dB (*n* = 14, Benkafadar et al., 2019). By comparison, in the considerably larger reference cohort of the present study, we observed a larger mean ABR threshold range of 63 dB in juvenile mice (1.5 months, *n* = 84) in contrast to a smaller range of 45 dB in adult mice (6 months, *n* = 22). The variance of ABR thresholds within age groups were found to be comparable, for example, at 8 and 16 kHz, mean SD of ABR threshold were estimated at 18.7 dB (*n* = 14, Benkafadar et al., 2019) compared with 19.8 dB (*n* = 22) in the present study. While further comparisons in the literature were limited due to the few studies describing age-related auditory function in the SAMP8 mice (Marie et al., 2017), previous and current data underline the functional variability in the SAMP8 strain, which needs to be taken into account in the experimental design, for example, for investigating age-related changes in auditory function or for evaluating therapeutic measures in long-term studies.

The overall progression of threshold loss over age in SAMP8 mice is comparable to other mouse models, but occurs within a shorter time scale (Fetoni et al., 2011). One of the most commonly used models is the C57BL/6J mouse, which presents a progressive high-frequency hearing loss from as early as 2 months of age (Li and Borg, 1991; Zheng et al., 1999) accompanied by broad degeneration of cochlear structures (Ison et al., 2007). While C57BL/6J mice already demonstrate a significant hearing loss in high frequencies (>20 kHz) starting at 2 months of age, low frequencies (≤8 kHz) first start to palpably deteriorate at 6 months of age with significant increase at 9–10 months of age (Li and Borg, 1991). Even though the audiometric progression in C57BL/6J mice also mimics the typical situation in human ARHL, this is observed over a course of 10 months or longer. By contrast, the SAMP8 strain exhibits a more rapidly progressing, significant hearing loss for a wide range of frequencies over the course of 6 months (see Figure 2). With regards to cytologic changes, an age-dependent progressive loss of both IHCs and OHCs was observed between 3 and 7 months of age in C57BL/6J, while loss of spiral ganglion neurons (SGNs) was evident by 7 months of age (Hequembourg and Liberman, 2001). By comparison, loss of sensory hair cells in SAMP8 can be observed at 1 month of age and degeneration of SGNs occurs already by 3 months of age (Menardo et al., 2012). Furthermore, the underlying cause behind the early onset of ARHL in C57BL/6J has been pinpointed to a mutation in the cadherin 23 gene (*Cdh23*), which encodes a cell component of the hair cell tip link (Noben-Trauth et al., 2003; Keithley et al., 2004). Given that ARHL is presumably influenced by multiple factors, with growing evidence pointing to oxidative stress as one of the main risk factors (Someya and Prolla, 2010; Menardo et al., 2012; Han and Someya, 2013; Benkafadar et al., 2019), the SAMP8 strain may prove to be a more appropriate and less time-consuming model of ARHL for several study questions.

The normal aging CBA/J mouse strain carries an *Ahl* resistant allele and does not develop premature hearing loss. Until the age of 1 year, this animal model presents almost no change in auditory thresholds (Zheng et al., 1999; Frisina and Zhu, 2010; Ohlemiller et al., 2010). The hearing sensitivity starts to decline in the high frequency region at the age of 19 months with a parallel loss of OHCs in the apical half of the cochlea (Ohlemiller et al., 2010), which is comparable to human cochlear aging (Wu et al., 2020). Despite the longer time scale of hearing loss progression in the normal aging CBA/J mouse strain, the variance of ABR thresholds has been shown to noticeably increase with age for low and high frequencies in the range 3–48 kHz (Frisina and Zhu, 2010), which is consistent with our observations in the SAMP8 strain over a shorter time scale.

In summary, compared with the C57BL/6J or the CBA/J strain, an accelerated senescence process, shorter lifespan, earlier onset and more rapid progression of age-related pathological phenotypes arguably make the SAMP8 mouse a useful animal model to study ARHL pathophysiology in a time frame that is permissive to animal experimentation. The large variability observed in auditory function needs to be taken into account in the experimental design and analysis. In future studies, predictive biomarkers should be investigated in order to allow better control and criteria selection of experimental SAMP8 groups.

### Auditory Threshold Increase Due to OHC or IHC Loss Over Age

Auditory thresholds are known to provide a sensitive measure of OHC function given their essential role in the lower dynamic range of the cochlear amplifier (Liberman and Kiang, 1978; Liberman and Beil, 1979; Dallos, 2008). The loss of the cochlear amplification has been shown to result in a CAP threshold shift of up to 30–40 dB in rats exposed to the ototoxic agent styrene (Chen et al., 2008), whose primary target are the OHCs. Their results further suggested that the resulting permanent threshold shift increases by approximately 6 dB per 10% OHC loss (Chen et al., 2008). In the present study, mean cytocochleograms showed an OHC loss first observed in young adult mice starting in the middle region and progressing toward the basal region (see Figure 5). With advancing age, a further increased OHC loss up to 40% could be observed. Our results showed an increase of the threshold shift in the high-frequency range by approximately 7.5 dB per 10% OHC loss, which generally corresponds to the observation of Chen et al. (2008). Although these results confirm OHC loss as one predictor of ABR threshold increase over age in SAMP8 mice, the observed threshold variability could not be explained by OHC loss alone, since OHC loss was comparable between mice allocated to the lower and upper quartiles (see Figure 6B) despite the persisting ABR threshold difference between these groups (see Figure 6A).

Human ARHL is best predicted by the loss of OHCs and IHCs, strongly suggesting sensory presbycusis as the predominant type of ARHL in humans (Wu et al., 2020). While the observed OHC loss in the SAMP8 mouse appears to be one predictor for age-related ABR threshold increase, this could not be confirmed for IHC loss. Despite the ABR threshold difference between the lower and upper quartiles in the experimental cohort, the loss of IHCs remained rather negligible for all age groups (see Figure 6B). A recent report showed that the selective loss of 70% of IHCs in a chinchilla model did not significantly change ABR thresholds (Jock et al., 1996; El-Badry and McFadden, 2009). Furthermore, in chinchillas, the selective IHC damage reduced the amplitude of CAP but the thresholds obtained from the higher brain regions, e.g., the inferior colliculus attributed to generating the ABR wave IV (Melcher and Kiang, 1996), remained unaffected (El-Badry and McFadden, 2007). The treatment with carboplatin, an ototoxic drug that selectively damages the IHC along the entire cochlea length in chinchillas, presented a negligible increase in threshold shift until the IHC loss exceeded 80% (Lobarinas et al., 2013). These studies indicate that ABR threshold shifts may not be influenced by the loss of IHC. In the past decade, multiple studies have suggested that afferent synapses may be the most vulnerable structures demonstrating a degeneration of synaptic connections prior to IHC loss (Stamataki et al., 2006; Sergeyenko et al., 2013; Wu et al., 2019). This preceding degeneration of synapses, known as “hidden hearing loss,” has been suggested to affect the ability to understand speech in noise (Badri et al., 2011; Vermiglio et al., 2012). The effect of afferent fiber loss on ABR thresholds has been previously shown to be negligible, e.g., in mice and guinea pigs no changes could be observed in ABR thresholds despite a significant loss of approximately 50% of afferent fibers (Kujawa and Liberman, 2009; Lin et al., 2011b).

In the SAMP8 model of ARHL, OHC loss has been previously shown to predominate age-related sensory degeneration (Menardo et al., 2012), which was confirmed in the present study as a secondary histopathological predictor for ABR thresholds. By contrast, IHC loss was not observed at least up to 7 months of age, while further progression of age can also lead to a delayed but progressive loss of IHCs (Menardo et al., 2012), which is similar to the human situation (Wu et al., 2020).

### Altered KCNQ4 and KCNQ1 Expressions as Potential Predictors of Hearing Loss

The premature SAMP8 senescence causing early presbycusis was linked to altered levels of antioxidant enzymes and decreased activity of complexes I, II, and IV, which in turn lead to chronic inflammation and triggering of apoptotic cell death pathways (Menardo et al., 2012). Before the premature onset of hearing loss, SAMP8 cochleae already displayed an early increase in ROS levels and downregulation of antioxidant enzymes (Menardo et al., 2012; Benkafadar et al., 2019). The levels of prooxidant molecules increased concomitantly with the degradation of hearing in SAMP8, while the deficit in antioxidants remained relatively stable until 6 months of age (Benkafadar et al., 2019). Given that an age-related progression of OHC loss was observed in the SAMP8 mice, we hypothesized that the threshold variability in SAMP8 mice might be due to a different progression of OHC loss in this subgroup thus creating this observed bifurcation of SAMP8 mice with ABR thresholds allocated to the lower or upper quartiles in each age group. Surprisingly, we could not observe differences in OHC loss between the two SAMP8 subgroups (see Figure 6B). Although our results indicated OHC loss as one predictor for ABR threshold increase over age, it does not appear to explain the variability of threshold progression between these subgroups.

The survival of OHCs is dependent on the hair cell conductance current which is carried by K^+^ (Spicer and Schulte, 1991, 1996; Schulte and Steel, 1994). This current drives the electromotility of OHCs (Ashmore and Gale, 2004) with KCNQ4 maintaining the OHC receptor potential (Kubisch et al., 1999a, b; Beisel et al., 2000; Kharkovets et al., 2000; Nouvian et al., 2003; Oliver et al., 2003; Holt et al., 2007). The ultrafast electromechanical gating of KCNQ4 in murine cochlear OHCs have been shown to develop with hearing as channel density in the basal pole of OHCs (Perez-Flores et al., 2020), while impaired membrane expression of KCNQ4 lead to progressive hearing loss (Jentsch, 2000a, b; Kharkovets et al., 2006; Gao et al., 2013; Carignano et al., 2019). In particular, the loss of KCNQ4 in the membrane of OHCs has been linked to a chronic depolarization, possibly increasing Ca^2+^ influx and causing the subsequent degeneration of OHCs due to chronic cellular stress (Ruttiger et al., 2004). Carignano et al. (2019) showed in a KCNQ4 knock-out (KO) mouse that the number of OHCs slowly decreased at a young age with increasing cell loss up to complete degeneration at oldest ages. Based on these previous observations, we hypothesized that diminished levels of KCNQ4 expression in OHCs could be an underlying cause of the observed ABR threshold variability in SAMP8 mice. KCNQ4 membrane expression averaged over three cochlear turns appeared to be reduced in mice with ABR thresholds allocated to the upper quartile (see Figure 8A). The persistent and increasing differences found between the lower and upper quartiles in KCNQ4 expression over age in the middle and midbasal turns, respectively (see Figure 9A), was further found to be congruent with the persistent ABR threshold difference observed over age between these groups, with larger differences in the high-frequency range (see Figure 6A).

Another essential component for maintaining auditory function is the recycling of K^+^ from OHCs to the endolymph. To facilitate the electromotility of OHCs, a high K^+^ concentration in the endolymph has to be maintained (Nin et al., 2016). One major component of the K^+^ recycling circuit is the voltage-dependent K^+^ channel KCNQ1, which is located in the marginal cells of the SV. KCNQ1 is responsible for the secretion of K^+^ to the endolymph and thereby generating and maintaining the EP (Wangemann et al., 1995; Wangemann, 2006). Loss-of-function mutations have been shown to impair K^+^ secretion into the endolymph leading to a defect in endolymph production accompanied by a collapse of Reissner’s membrane (Casimiro et al., 2001). Another study could observe a decrease in KCNQ1 causing SV atrophy in aged C57BL6/J with notable hearing loss (Yang et al., 2013). We thus hypothesized that diminished levels of KCNQ1 expression in the SV could be another underlying cause of the observed ABR threshold variability in SAMP8 mice. While mice allocated to either quartile generally did not show a correlation between KCNQ1 expression and age, a persistent difference over age may be interpreted between the lower and upper quartiles in the midbasal turn (see Figure 9B).

KCNQ4 and KCNQ1 as K^+^ channels generally possess high energy requirements for continuous recycling processes to enable a proper membrane expression, suggesting them as one of the primary targets of ROS accumulations (Peixoto Pinheiro et al., 2020). Furthermore, ROS-mediated modification of K^+^ channels can induce alterations of the activity of signaling mechanisms causing changes in channel activity or channel gene expression (Ruppersberg et al., 1991; Matalon et al., 2003; Cai and Sesti, 2009). As mentioned above, the premature senescence of SAMP8 mice causing early presbycusis was previously linked to altered levels of antioxidant enzymes and an accumulation of ROS. An impaired membrane expression and a disrupted KCNQ4 conductance causes progressive sensorineural hearing loss (Gao et al., 2013). Furthermore, cells in the SV have been shown to contain large numbers of mitochondria, predisposing them to an increased vulnerability to oxidative stress (Nakazawa et al., 1995; Spicer and Schulte, 2002). Consequently, reduced membrane expressions of KCNQ4 and KCNQ1 caused by ROS accumulation are suggested as potential predictors of the observed ABR threshold variability in SAMP8 mice, feasibly preceding OHC loss over age.

### Other Potential Phenotypic Predictors of Hearing Loss

Although OHC and IHC loss have been demonstrated as the most important histologic predictors of human ARHL in both high- and low-frequency regions, there remain age-related effects that have not been identified in the cytohistological analysis of the human cochlea (Wu et al., 2020). These uncaptured effects may be found in the phenotype of surviving sensory hair cells or other functionally relevant structures such as the SV. As the loss of sensory hair cells and reduction in hearing function was not influenced by a single cause, we cannot exclude other cellular or phenotypic predictors that could have an effect on the progression of the ABR threshold variability in SAMP8 mice (Wangemann et al., 1995; Vetter et al., 1996).

The degeneration of SGNs can be observed both in humans and animal models of ARHL (Keithley and Feldmann, 1979; Makary et al., 2011; Viana et al., 2015). Previous studies have shown that the neuronal loss precedes the degeneration of IHCs (Viana et al., 2015; Wu et al., 2019). However, the loss of neurons did not lead to elevated ABR thresholds (Schuknecht and Woellner, 1955; Kujawa and Liberman, 2009). While Menardo et al. (2012) have observed an SGN loss of about 20% by 6 months of age in SAMP8 mice (here we describe mice up to 7 months of age), it was previously reported that even a significant loss of 50% SGN did not affect ABR thresholds in mice and guinea pigs (Kujawa and Liberman, 2009; Lin et al., 2011b). Therefore, loss of SGNs appears to play a minor role in the prediction of threshold elevation, but very likely contributes to significant decrease in speech discrimination, especially in noisy environments (Schuknecht and Woellner, 1955; Kujawa and Liberman, 2009; Wu et al., 2019).

Another potential predictor is the OHC motor protein prestin. Liberman et al. (2002) observed an increase of ABR thresholds in prestin KO animals compared with wild type. In addition, the absence of OHC electromotility due to prestin KO resulted in a loss of cochlear sensitivity of approximately 40–60 dB (Liberman et al., 2002). In the F344 rat, a disruption of prestin has been reported in aged OHCs and it was thus suggested that ROS accumulation during aging may affect prestin through protein oxidation (Chen et al., 2009; Syka, 2010). However, it remains unclear to which degree the disruption of prestin relates to OHC somatic electromotility and motor function sensitivity (Cheatham et al., 2005).

## Conclusion

The present study supported our initial hypothesis that the large variability of threshold loss over time could be explained by a bifurcation into two subgroups, where these SAMP8 mice constituted the lower and upper quartiles of the threshold distribution, respectively. Surprisingly, the progression of threshold variability could not be linked to a parallel ongoing loss of OHCs. By contrast, OHC loss appeared to be preceded by an altered phenotype of OHCs linked to KCNQ4 membrane expression and possibly an altered phenotype of the SV linked to KCNQ1, which appeared to be decreased in mice with ABR thresholds allocated to the upper quartile with higher ABR thresholds when compared to age-matched mice in the lower quartile. We suggest that the integrity of KCNQ4 and KCNQ1 channels has to be considered as a possibly decisive step for elevated hearing loss in SAMP8 mice that precedes OHC loss. The observed phenotypic change may underlie age-related effects of ARHL in SAMP8 mice preceding irreversible OHC loss due to accumulated metabolic challenges with age. Considering this, increase of antioxidant activity, decrease of ROS production, or pharmaceutical targeting of K^+^ channels could be promising approaches to decelerate or prevent ARHL before the inevitable progression of hair cell loss.

## Data Availability Statement

The raw data supporting the conclusions of this article will be made available by the authors, without undue reservation, to any qualified researcher.

## Ethics Statement

The animal study was reviewed and approved by the veterinary care unit of the University of Tübingen and by the regional Animal Care and Ethics Committee (Regierungspräsidium Tübingen).

## Author Contributions

BP, YA, MK, and HL contributed to conceptualization and writing. BP and YA conducted the experiments and performed statistical analysis. YA, MK, MM, and HL contributed to supervision and interpretation of data. All authors contributed to revision, editing and approved the final version of the manuscript.

## Conflict of Interest

HL is Scientific Founder and a member of the board of directors of Acousia Therapeutics (Tübingen, Germany). The remaining authors declare that the research was conducted in the absence of any commercial or financial relationships that could be construed as a potential conflict of interest.

## Publisher’s Note

All claims expressed in this article are solely those of the authors and do not necessarily represent those of their affiliated organizations, or those of the publisher, the editors and the reviewers. Any product that may be evaluated in this article, or claim that may be made by its manufacturer, is not guaranteed or endorsed by the publisher.
