# Supplementary material for: Auditory Threshold Variability in the SAMP8 Mouse Model of Age-Related Hearing Loss: Functional Loss and Phenotypic Change Precede Outer Hair Cell Loss
Source: Front Aging Neurosci. 2021 Aug 2;13:708190. doi: 10.3389/fnagi.2021.708190 (PMC8366269; doi:10.3389/fnagi.2021.708190)
Supplement: Supplementary file 1 [file Data_Sheet_1.docx]

Supplementary Material

# Supplementary Data

**
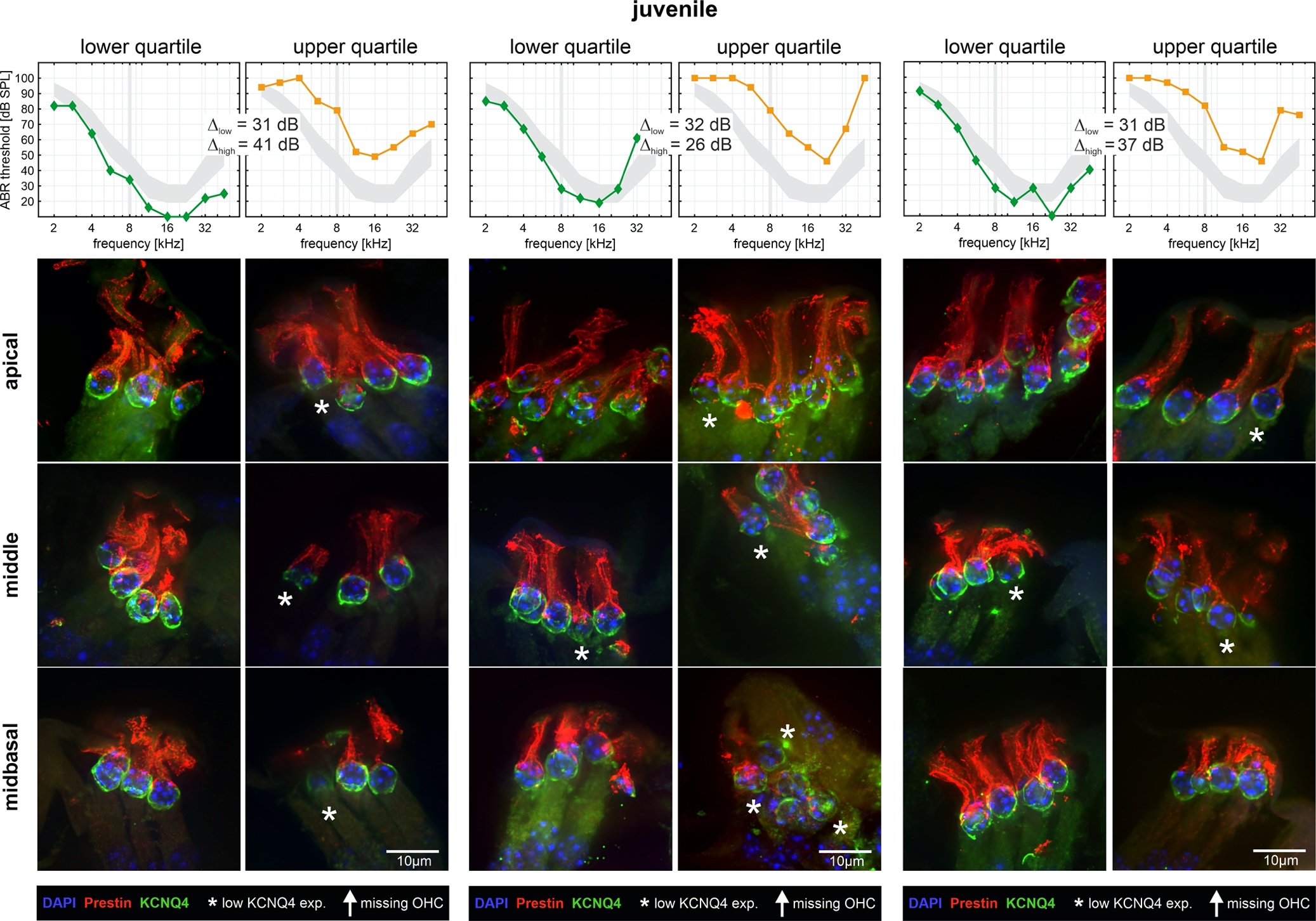
**

**Supplementary Figure 1:** Comparison of auditory brainstem response (ABR) thresholds and corresponding cochlear cross-sections of juvenile mice in the lower (green) and upper (orange) quartiles. ABR threshold differences were calculated for the low- (≤ 8 kHz, Δ_low_) and high-frequency (> 8 kHz, Δ_high_) range, respectively. Cochlear cross-sections in apical, middle, and midbasal turns were stained with KCNQ4 (green), prestin (red) for the outer hair cell (OHC) motor protein, and DAPI (blue) for cell nuclei. Asterisks indicate low KCNQ4 surface expression, arrows highlight missing OHCs, and a scale bar of 10 µm is shown.

**
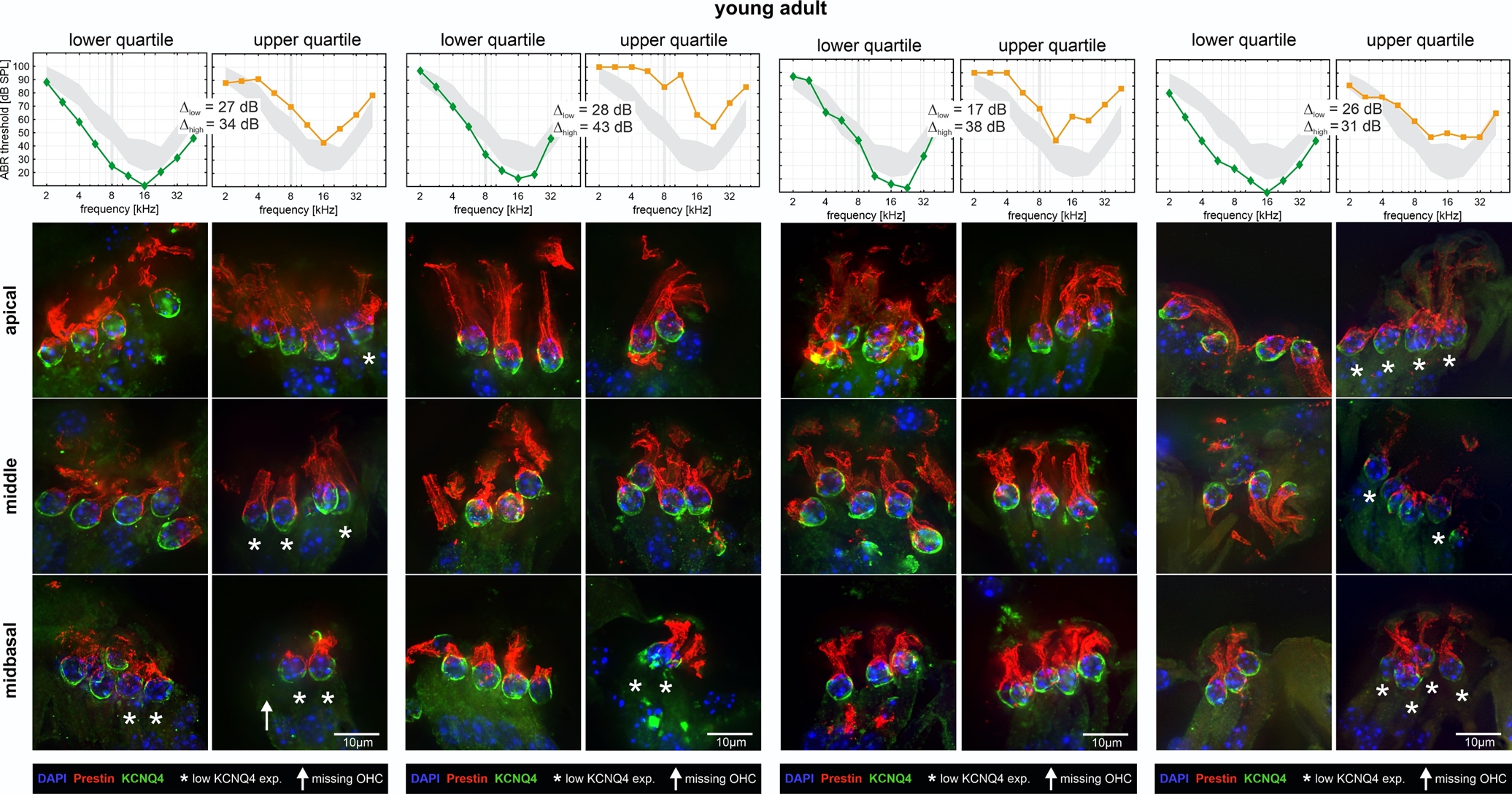
**

**Supplementary Figure 2**: Comparison of auditory brainstem response (ABR) thresholds and corresponding cochlear cross-sections of young adult mice in the lower (green) and upper (orange) quartiles. ABR threshold differences were calculated for the low- (≤ 8 kHz, Δlow) and high-frequency (> 8 kHz, Δhigh) range, respectively. Cochlear cross-sections in apical, middle, and midbasal turns were stained with KCNQ4 (green), prestin (red) for the outer hair cell (OHC) motor protein, and DAPI (blue) for cell nuclei. Asterisks indicate low KCNQ4 surface expression, arrows highlight missing OHCs, and a scale bar of 10 µm is shown.

**
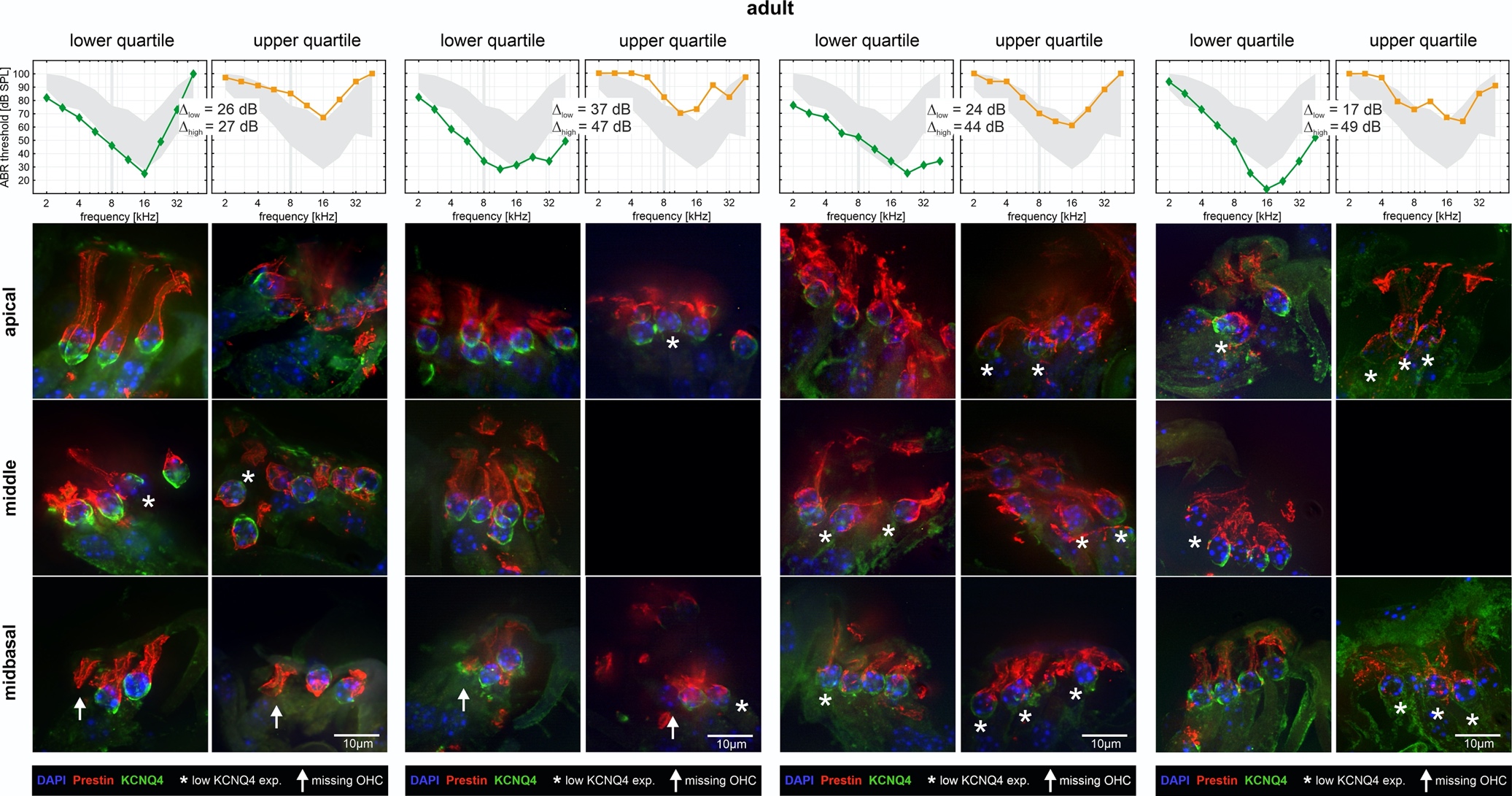
**

**Supplementary Figure 3**: Comparison of auditory brainstem response (ABR) thresholds and corresponding cochlear cross-sections of adult mice in the lower (green) and upper (orange) quartiles. ABR threshold differences were calculated for the low- (≤ 8 kHz, Δlow) and high-frequency (> 8 kHz, Δhigh) range, respectively. Cochlear cross-sections in apical, middle, and midbasal turns were stained with KCNQ4 (green), prestin (red) for the outer hair cell (OHC) motor protein, and DAPI (blue) for cell nuclei. Asterisks indicate low KCNQ4 surface expression, arrows highlight missing OHCs, and a scale bar of 10 µm is shown. Due to cryosectioning difficulties, the cross-section in the middle turn of the mouse in the upper quartile of the second (fourth column) and the fourth pairwise comparison (eighth column) was excluded.


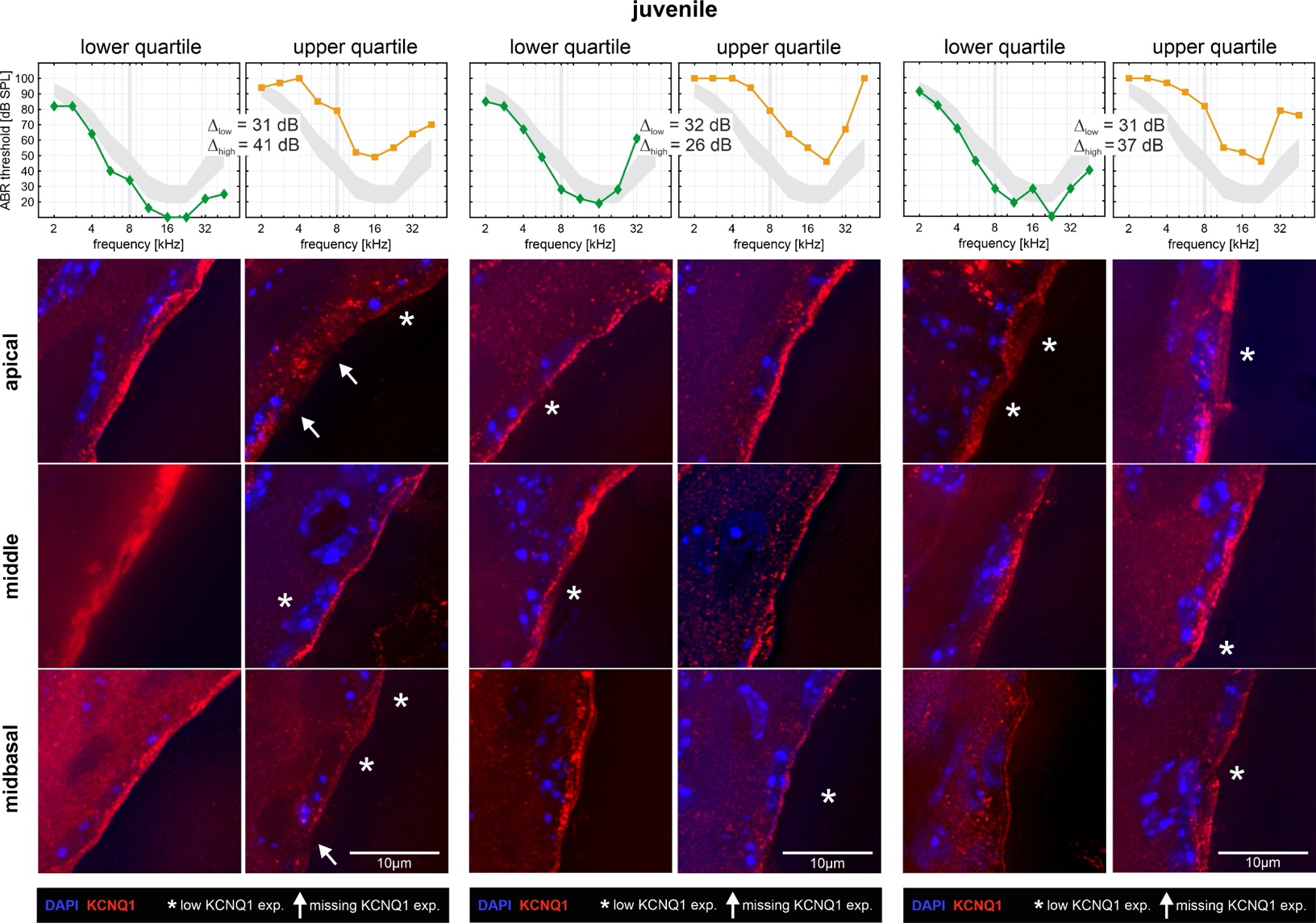


**Supplementary Figure 4**: Comparison of auditory brainstem response (ABR) thresholds and corresponding cochlear cross-sections of juvenile mice in the lower (green) and upper (orange) quartiles. ABR threshold differences were calculated for the low- (≤ 8 kHz, Δlow) and high-frequency (> 8 kHz, Δhigh) range, respectively. Cochlear cross-sections in apical, middle, and mid-basal turns were stained with KCNQ1 (red), a potassium channel expressed in the stria vascularis, and DAPI (blue) for nuclei. Asterisks indicate low KCNQ1 surface expression (exp.), arrows highlight the absence of KCNQ1, and a scale bar of 10 µm is shown.

**
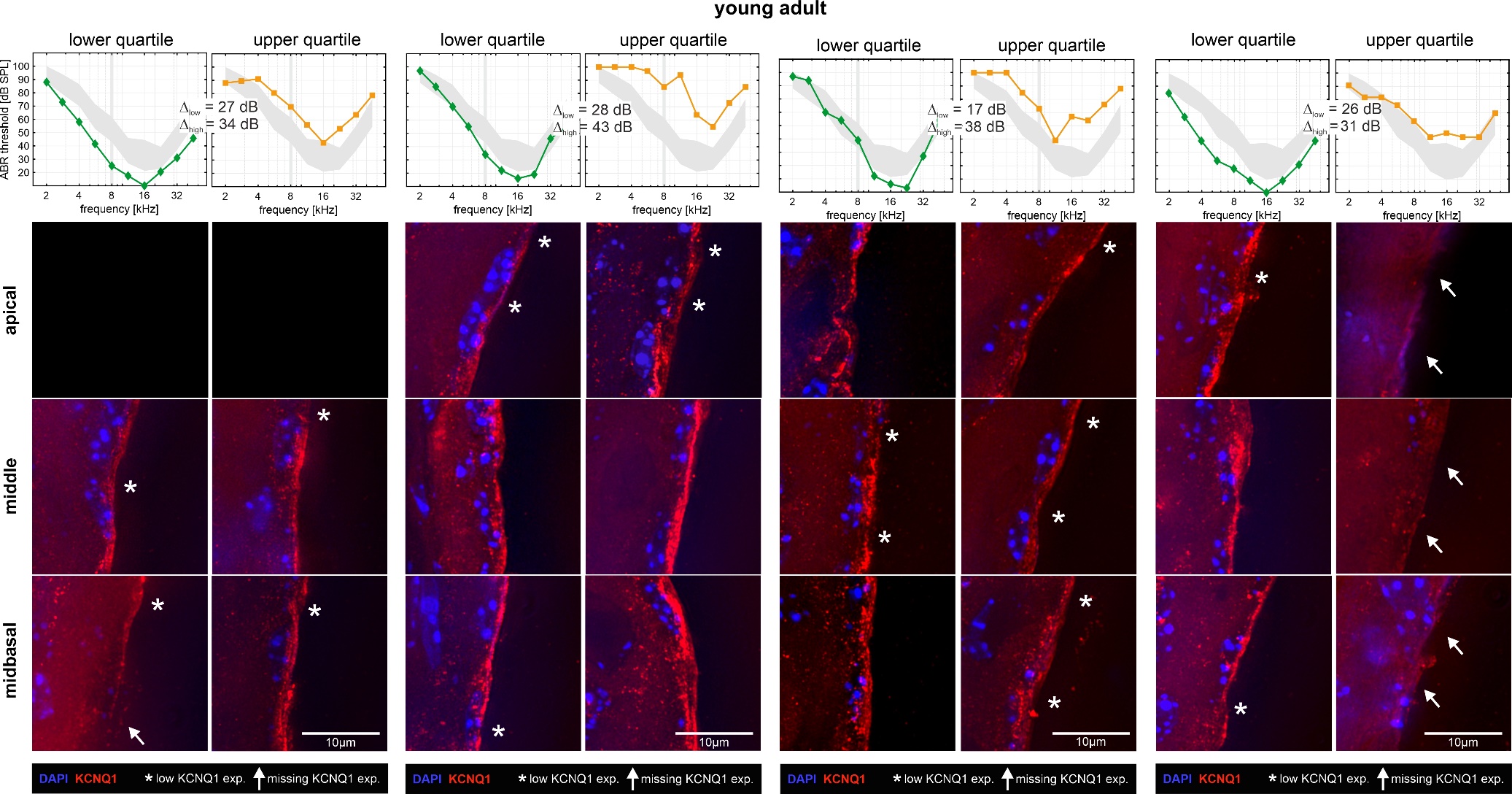
**

**Supplementary Figure 5**: Comparison of auditory brainstem response (ABR) thresholds and corresponding cochlear cross-sections of young adult mice in the lower (green) and upper (orange) quartiles. ABR threshold differences were calculated for the low- (≤ 8 kHz, Δlow) and high-frequency (> 8 kHz, Δhigh) range, respectively. Cochlear cross-sections in apical, middle, and mid-basal turns were stained with KCNQ1 (red), a potassium channel expressed in the stria vascularis, and DAPI (blue) for nuclei. Asterisks indicate low KCNQ1 surface expression (exp.), arrows highlight the absence of KCNQ1, and a scale bar of 10 µm is shown. Due to cryosectioning difficulties, the cross-section in the apical turn of the mouse in the upper and lower quartile of the first pairwise comparison (first and second column) was excluded.

**
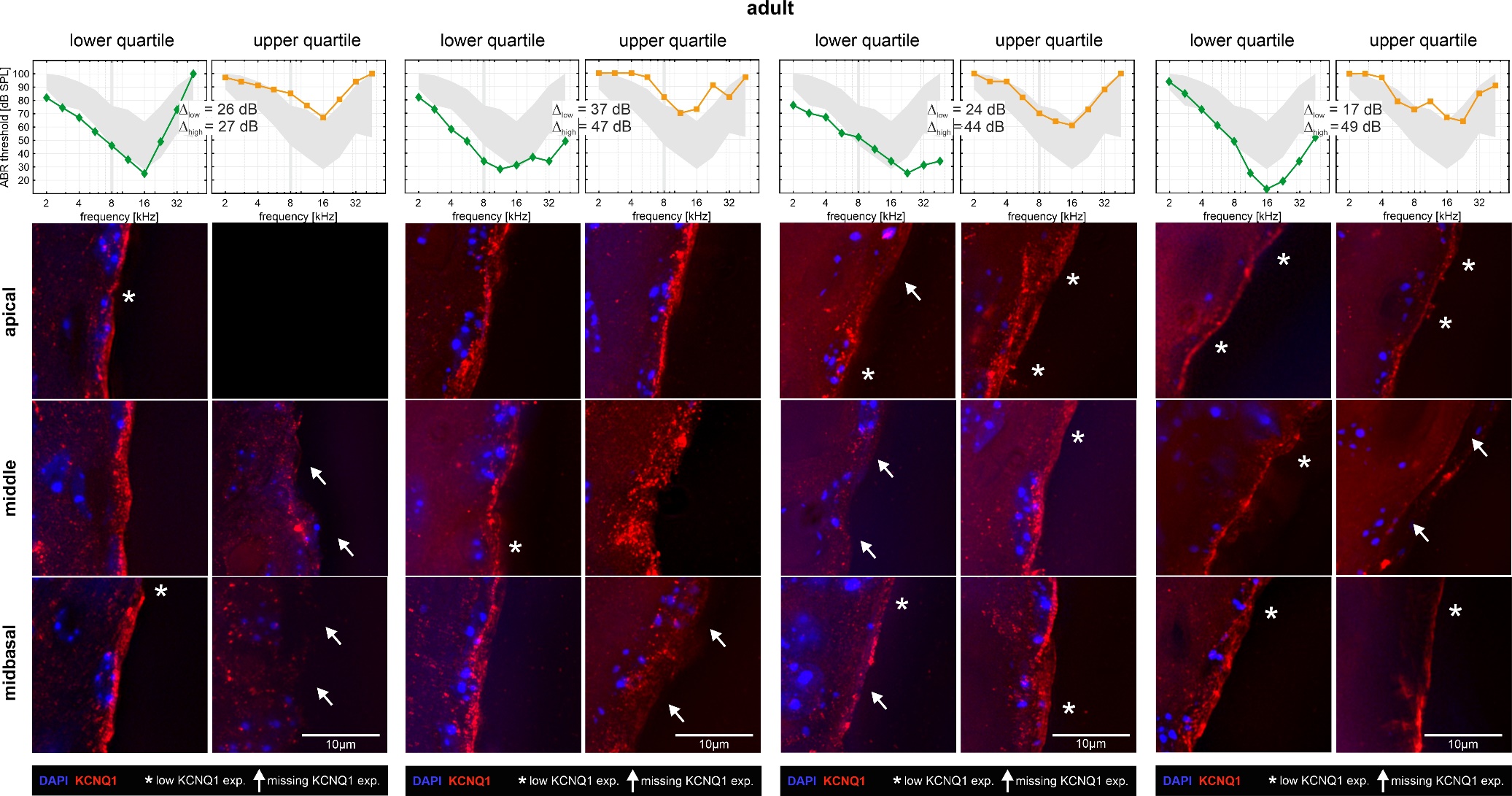
**

**Supplementary Figure 6**: Comparison of auditory brainstem response (ABR) thresholds and corresponding cochlear cross-sections of adult mice in the lower (green) and upper (orange) quartiles. ABR threshold differences were calculated for the low- (≤ 8 kHz, Δlow) and high-frequency (> 8 kHz, Δhigh) range, respectively. Cochlear cross-sections in apical, middle, and mid-basal turns were stained with KCNQ1 (red), a potassium channel expressed in the stria vascularis, and DAPI (blue) for nuclei. Asterisks indicate low KCNQ1 surface expression (exp.), arrows highlight the absence of KCNQ1, and a scale bar of 10 µm is shown. Due to cryosectioning difficulties, the cross-section in the apical turn of the mouse in the lower quartile of the first pairwise comparison (second column) was excluded.
